# Role of oxygen vacancies in ferroelectric or resistive switching hafnium oxide

**DOI:** 10.1186/s40580-023-00403-4

**Published:** 2023-12-01

**Authors:** Jaewook Lee, Kun Yang, Ju Young Kwon, Ji Eun Kim, Dong In Han, Dong Hyun Lee, Jung Ho Yoon, Min Hyuk Park

**Affiliations:** 1 Department of Materials Science and Engineering and Inter-University Semiconductor Research Center, College of Engineering, Seoul National University, Gwanak-Ro 1, Gwanak-Gu, Seoul, 08826 Republic of Korea; 2 Electronic Materials Research Center, Korea Institute of Science and Technology (KIST), Seoul, 02791 Republic of Korea; 3 Research Institute of Advanced Materials, Seoul National University, Gwanak-Ro 1, Gwanak-Gu, Seoul, 08826 Republic of Korea

**Keywords:** HfO_2_, Ferroelectricity, Resistive switching, Semiconductor, Memory device

## Abstract

HfO_2_ shows promise for emerging ferroelectric and resistive switching (RS) memory devices owing to its excellent electrical properties and compatibility with complementary metal oxide semiconductor technology based on mature fabrication processes such as atomic layer deposition. Oxygen vacancy (V_o_), which is the most frequently observed intrinsic defect in HfO_2_-based films, determines the physical/electrical properties and device performance. V_o_ influences the polymorphism and the resulting ferroelectric properties of HfO_2_. Moreover, the switching speed and endurance of ferroelectric memories are strongly correlated to the V_o_ concentration and redistribution. They also strongly influence the device-to-device and cycle-to-cycle variability of integrated circuits based on ferroelectric memories. The concentration, migration, and agglomeration of V_o_ form the main mechanism behind the RS behavior observed in HfO_2_, suggesting that the device performance and reliability in terms of the operating voltage, switching speed, on/off ratio, analog conductance modulation, endurance, and retention are sensitive to V_o_. Therefore, the mechanism of V_o_ formation and its effects on the chemical, physical, and electrical properties in ferroelectric and RS HfO_2_ should be understood. This study comprehensively reviews the literature on V_o_ in HfO_2_ from the formation and influencing mechanism to material properties and device performance. This review contributes to the synergetic advances of current knowledge and technology in emerging HfO_2_-based semiconductor devices.

**Graphical Abstract:**

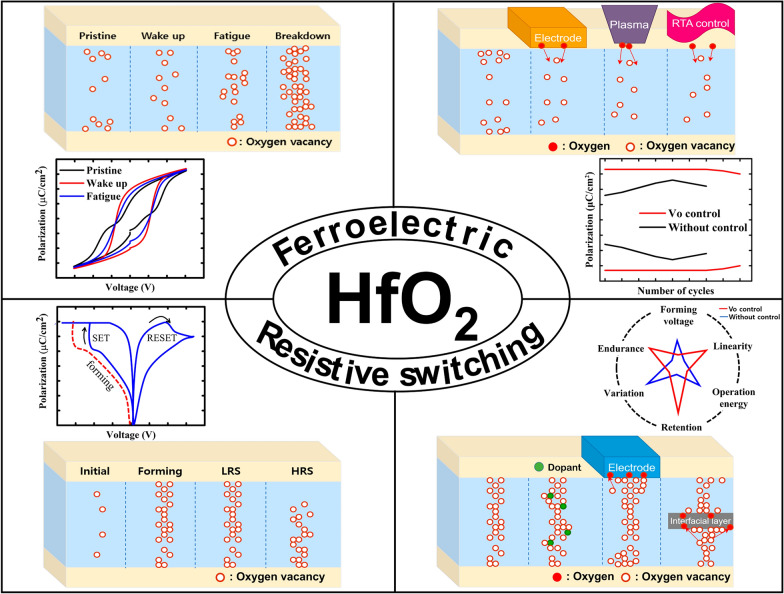

## Introduction

Among various candidate metal oxides, HfO_2_ has shown the most promise for emerging semiconductor devices, including ferroelectric and RS memories. Table 1 compares the various properties of insulating metal oxides frequently used or potentially considered in the semiconductor industry. HfO_2_ has an electrical bandgap as high as 5.0–6.0 eV and a sufficiently high crystallographic-structure-dependent dielectric constant of 20–40. Although the bandgap of HfO_2_ is lower than those of SiO_2_ (approximately 9 eV) and Al_2_O_3_ (approximately 7 eV), it can form a significantly high Schottky barrier upon contact with high-work-function metals. Moreover, the dielectric constant of monoclinic or amorphous HfO_2_ is approximately 4–5 times higher than that of SiO_2_. This value can be further enhanced with the higher-k metastable phases such as tetragonal, cubic, and orthorhombic phases. Therefore, HfO_2_ is suitable for insulating high-k materials while preventing direct electrical conduction by enabling the penetration of a relatively high fraction of electric field to the neighboring materials (e.g., the semiconductor channel in metal oxide semiconductor field effect transistors, MOSFETs). Hence, HfO_2_ has emerged as the most frequently and early considered high-k material among numerous candidates.

**Table 1:** Comparison of properties of various metal oxides (potentially) utilized in semiconductor processes

Property	HfO_2_	SiO_2_	Al_2_O_3_	ZrO_2_	TiO_2_
Bandgap [eV]	~ 5.5 [229]	~ 9 [230]	~ 7 [231]	5–7 [232]	~ 3.2 [233]
Dielectric constant	~ 16 (Monoclinic) ~ 29 (Cubic) ~ 70 (Tetragonal) [234]	3.9 [235]	9 [235]	~ 20 (Monoclinic) ~ 37 (Cubic) ~ 47 (Tetragonal) [234]	~ 31 (Anatase) ~ 114 (Rutile) [236]
Formation enthalpy [kJ/mol]	–1,113.20 [140]	–320.7 [237]	–1,573 [238]	–1,097.46 [239]	–891.2 [238]
M–O bond length [Å]	2.2 [235]	1.7 [235]	1.9 [235]	2.2 [235]	2 [235]
M–O bonding energy [kJ/mol]	801 ± 13	799.6 ± 13.4	542	766.1 ± 10.6	672 ± 9

HfO_2_ became the first material to replace SiO_2_ as the gate insulator in a commercial semiconductor chipset with its adoption in Intel’s Penryn processor in 2007 via atomic layer deposition (ALD). The ALD technique is suitable for homogeneous and uniform film deposition even on complex nanostructures. Moreover, the technique controls thickness with atomic-level accuracy, enabling ferroelectricity or RS in nanoscale semiconductor devices for ultra-large-scale integrated circuits. Various Hf precursors and reactants were adopted to deposit ferroelectric or RS HfO_2_ as well as conventional gate-insulating HfO_2_ in MOSFETs [1–4].

**Fig. 1:**
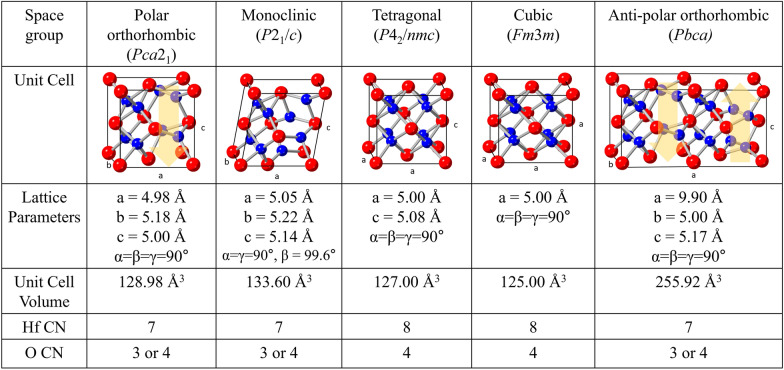
Frequently observed crystallographic phase of HfO_2_, presenting the lattice parameters, unit cell volume, and coordination number of Hf and oxygen ions in each phase

The characteristic electrical, physical, and chemical properties of HfO_2_ are based on their fluorite crystallographic structure. It is known that HfO_2_ exists in various polymorphs, including the monoclinic (*P*2_1_/*c*), tetragonal (*P*4_2_/*nmc*), orthorhombic (*Pca*2_1_ or *Pbca*), and cubic (*Fm-*3*m*) phases. The eight oxygen ions are located at the tetrahedral sites and have three or four ionic bonds with the surrounding metal ions, and the hafnium ions have seven or eight coordination numbers. The differences in the chemical bonding and the ensuing crystallographic structures are particularly important for the ferroelectric properties because ferroelectricity originates from broken centrosymmetry, which results in reversible spontaneous polarization states. Figure 1 shows the crystallographic structure of various polymorphs of HfO_2_. The cubic phase is the fluorite-structure-based crystallographic phase with the highest multiplicity and the largest number of symmetry elements. This phase is known to be stable when the temperature is higher than 2,573 K owing to its high configurational entropy [5]. All the other polymorphs can be constructed from the cubic phase by partially removing the symmetry elements of the cubic phase. The scope of this study does not permit a discussion on all possible polymorphs of HfO_2_. Hence, we only expound on the frequently observed crystallographic phases such as monoclinic, tetragonal, cubic, and *Pca*2_1_ and *Pbca* orthorhombic phases.

Given the coordination numbers of oxygen and hafnium ions, these polymorphs can be categorized into two groups, one comprising the monoclinic, and *Pca*2_1_ and *Pbca* orthorhombic phases and the other comprising the tetragonal and cubic phases. In the former group, the coordination number of hafnium ion is seven, whereas that of oxygen ions is three or four. In the latter group, the coordination number of hafnium ion is eight, whereas that of oxygen ion is four. When the lattice parameters and unit cell volumes of the polymorphs are considered, the monoclinic phase is distinct from the other phases with larger lattice parameters and beta angle higher than 90°. Therefore, the resulting unit cell volume is also considerably different (~ 3.6 to 6.9% larger). To summarize, the ferroelectric *Pca*2_1_ orthorhombic phase is similar to the monoclinic phase when considering the ionic coordination numbers, whereas it is similar to the tetragonal or cubic phase when considering the unit cell volumes and lattice angles.

Moreover, the *Pbca* orthorhombic phase is highly relevant to the *Pca*2_1_ orthorhombic phase. Materlik et al. [6] reported that inducing two opposite polarization states in *Pca*2_1_ orthorhombic phase unit cells generates the *Pbca* orthorhombic phase. Thus, the *Pbca* orthorhombic phase can be called antipolar phase, which is frequently considered for the origin of the antiferroelectric properties according to the classical Kittel’s model [7]. However, according to experimentally observed temperature-dependent changes in ferroelectric–paraelectric transitions, the antiferroelectricity in HfO_2_ is believed to originate from the reversible field-induced phase transition between the tetragonal and *Pca*2_1_ orthorhombic phases rather than that between the *Pbca* and *Pca*2_1_ orthorhombic phases [8, 9]. Interestingly, the antipolar *Pbca* orthorhombic phase has a lower free energy than the polar *Pca*2_1_ orthorhombic phase under almost every temperature and pressure range examined in previous computational simulations. Therefore, the formation of the *Pbca* phase is highly probable. Recently, it was elucidated that the formation of the electrical cycling driven formation of the *Pbca* phase is the crystallographic origin of low field fatigue observed in ferroelectric Hf_0.5_Zr_0.5_O_2_ (HZO) thin films [9]. Although this phenomenon was observed at room temperature, a similar phenomenon is expected over wide ranges of temperature and pressure, when small free-energy and configurational-entropy differences between the two orthorhombic phases arise from their crystallographic similarity. Thus, it is believed that the antipolar *Pbca* orthorhombic phase should be further studied to understand the complex nanoscale polymorphism of HfO_2_-based thin films.

Notably, other interesting non-centrosymmetric crystallographic phases of HfO_2_ were reported in theoretical and experimental works. The *Pmn*2_1_ orthorhombic phase is another interesting polar crystallographic phase with remarkably high spontaneous polarization and a small energy difference with the stable monoclinic or polar *Pca*2_1_ orthorhombic phase. However, to the best of the authors’ knowledge, this phase has not been experimentally observed [10]. Rhombohedral *R*3 or *R*3*m* phases are also other interesting polar phases, which have been observed in epitaxial HfO_2_-based ferroelectric thin films fabricated using pulsed laser deposition (PLD) [11]. However, the *Pca*2_1_ orthorhombic phase would be more focused on in this study.

The RS characteristics in HfO_2_ are less sensitive to polymorphism, unlike ferroelectricity that is deterministically affected by the crystallographic phase. Conversely, the degree of V_o_ formation in the different fluorite crystallographic structures of HfO_2_ strongly influences the RS characteristics. For example, there is a difference in the magnitude of initial electroforming voltage depending on the number of Hf–O bonds of a unit cell in the crystalline phase, as reported by S. U. Sharath et al. [12]. However, in the case of V_o_-mediated RS characteristics, both the crystallographic phase and stoichiometry or oxygen deficiency determined during the deposition or fabrication process can be considered significant factors influencing the intrinsic V_o_ defect. The electromigration or accumulation of V_o_ under an applied voltage and the consequent formation of a specific conductive pathway called the conductive filament (CF) should also be essentially considered. HfO_2_ has been studied as a RS material since its early research stage because of its CMOS compatibility and the absence of an oxygen-deficient subphase, such as the Magnéli phase in TiO_2_. In RS memory devices, the HfO_2_ layer is used as a switching layer between an inert and a reactive metal electrode, which illustrates the RS operation attributed to V_o_ dynamics. From these studies, it can be concluded that the RS characteristics can be controlled by adjusting the formation, migration, and accumulation of V_o_. In addition, it is easy to form the amorphous and crystalline phases, including the grain and grain boundary engineering, depending on the deposition and post-processing conditions, resulting in different properties according to each influence. The subsequent switching characteristics after electroformation are also strongly correlated with these above oxygen vacancy-related factors. Several outstanding properties of HfO_2_-based RS memory devices, such as large memory window, multi-level storage ability, low power consumption [13–15], and CMOS compatibility [16] with possible high-density or three-dimensional integration, have attracted a considerable research strong interest for their application in neuromorphic computing [17–19], in particular for the development of artificial synapses or neurons in neural networks. Most studies that reported V_o_-mediated RS behavior in HfO_2_ focused on V_o_-related factors and their relationships with electroformation and the subsequent switching performance to realize high-precision multilevel operation, and on the analog weight update in the synaptic application to exploit the resistance dynamics of memory devices. Similar to biological synapses, nonvolatile RS devices act as memory components, storing the strength of synaptic connections without power. However, for these RS devices to fully mimic synaptic behavior, they must meet specific requirements, including forming voltage, variation, switching behavior, switching speed, switching voltage, conductance modulation, endurance, and retention. As the V_o_ dynamics in an RS device significantly affect these multiple performance aspects, improving V_o_ controllability is crucial to accurately implement synaptic operations in artificial systems. In this review, we discuss several technical solutions to control the V_o_ dynamics for enhancing the performance of HfO_2_-based RS devices.

As mentioned earlier in this section, V_o_ might significantly influence the ferroelectricity and RS of HfO_2_, suggesting that a meticulous theory of the chemical, physical, and electrical properties of V_o_ and their behavior in HfO_2_ must be developed. Furthermore, from the similarity between the operating electric field and various pieces of experimental evidence, a comprehensive comparison and in the V_o_ effects on ferroelectricity and RS HfO_2_ would be helpful for synergistically advancing our knowledge on HfO_2_-based ferroelectric and RS memories. In this review, therefore, the physical/electrical effects, formation mechanism, and ferroelectricity and RS effects of V_o_ in HfO_2_-based thin films are discussed.

The structure of the review is as follows. In Chapter 2, the V_o_ formation, migration, and accumulation in HfO_2_ thin films are detailed as investigated in extant literature. In Chapters 3 and 4, the effects of V_o_ on ferroelectricity and RS in HfO_2_ thin films are reviewed, and the strategies to improve the ferroelectricity or RS are discussed based on the literature. In Chapter 5, V_o_-mediated ferroelectricity and RS behavior are expounded. Based on this, we provide strategies to engineer devices for ferroelectric and RS memory. Finally, the conclusions and future outlook are presented in Sect. 5.

## Oxygen vacancy formation, migration, and accumulation in HfO_2_

V_o_ is the most frequently observed intrinsic point defect in metal oxide thin films or bulk. The existence of V_o_ induces an imperfect bonding state in crystals with three-dimensional periodic arrays of atoms, ions, molecules, or their groups. Consequently, the enthalpy of materials increases with the increasing concentration of V_o_. However, the configurational entropy would decrease for a specific range of V_o_ concentration. Thus, there is an equilibrium V_o_ concentration which is strongly affected by temperature. Generally, the equilibrium vacancy concentration increases with increasing temperature because the impact of the entropy term on the Gibbs free energy is enhanced with temperature. Furthermore, once formed vacancies tend to be remained without oxygen providing atmosphere, so the high temperature processes during the material fabrication is critical for the V_o_ concentration. To summarize, thermodynamically, finite V_o_ should exist in metal-oxide thin films, and it is same for the fluorite-structure oxides such as hafnia and zirconia, which are frequently utilized in classical (MOSFET or dynamic random-access memory, DRAM) or emerging semiconductor devices (ferroelectric memories and memristors).

The existence of V_o_ strongly influences the physical/chemical properties of metal oxides. Electrostatically, the V_o_ form electron trap levels in the forbidden bandgap and contribute to the local conduction, which is generally considered harmful for the insulating layer [20]. For memristor applications, however, the filament formation by accumulated V_o_ is one of the RS mechanisms [21]. For the case of ferroelectric (Hf,Zr)O_2_-based films, V_o_ have been reported as one of the many factors affecting the polymorphism that produces ferroelectricity [22, 23]. Charged V_o_ are mobile under a high electric field (in the order of MV/cm), so their drift is considered a major parameter impacting the dynamic changes in performances of electronic devices with metal oxides.

This discussion highlights the importance of understanding the physics and chemistry of V_o_ in (Hf,Zr)O_2_-based materials for improving the performance of semiconductor devices, such as ferroelectric memories and memristors. In this section, the factors influencing their formation, such as deposition conditions, dopants, and electrode materials, are discussed. Subsequently, the observation of V_o_ migration through a transmission electron microscope (TEM) and their roles are addressed. Lastly, the aggregation of V_o_ and the underlying conduction mechanism are discussed.

### Factors affecting the formation of V_o_

#### Deposition condition

Various deposition techniques such as chemical solution deposition (CSD) [24–27], sputtering [28–31], PLD [32], and ALD [1] are used to deposit the (Hf,Zr)O_2_ film. In the CSD method, the precursor chemicals are dissolved in a solvent, and the solution is deposited on the substrate through spin or spray coating. The subsequent drying removes the solvent, resulting in an amorphous film [25]. The CSD is economical, suitable for mass production, and does not require a vacuum environment; however, it is difficult to decrease the concentration of impurities to extremely low levels required for modern CMOS technology. Various research groups have investigated CSD-grown HfO_2_-based thin films [24, 26, 27, 33, 34]. However, the effect of oxygen vacancies, which is the main topic of this review, has not been studied frequently in CSD-grown HZO thin films compared to that grown using other deposition techniques. Starschich et al. demonstrated that the drift of oxygen vacancies is the origin of both the wake-up effect and RS in CSD grown ferroelectric HZO thin films [35], indicating that understanding the effects of oxygen vacancies is critical for both ferroelectric and resistive memories based on (Hf,Zr)O_2_ thin films.

Sputtering is a physical vapor deposition (PVD) technique in which ionized gas molecules in plasma are accelerated toward the target to induce ion bombardment. Subsequently, the atoms in the target eject and fly from the target to the substrate. Sputtering offers a low-pressure deposition process favorable for fabricating films with a low concentration of impurities such as C, H, and N [31]. Likewise, PLD is a PVD technique wherein a pulsed laser beam is focused on a target, resulting in the formation of a plasma plume and the growth of the film on the substrate [36]. In PVD techniques, the ratio of the O_2_/Ar gas flow and pressure can affect the concentration of V_o_ and the film properties [29, 31, 37]. Jaszewski et al. studied the effect of the O_2_/Ar gas flow ratio on the concentration of V_o_ in the HfO_x_ film using reactive sputtering. The value of x in HfO_x_ obtained from the low-loss electron energy loss spectroscopy (EELS) spectra increased from 1.51 to 1.68 as the proportion of O_2_ gas in the plasma increased from 7.4 to 8.0%. Thus, the amount of V_o_ in the HfO_2_ film decreases with an increase in the O_2_ gas proportion in the plasma during reactive sputtering. Song et al. suggested that, because of the high-energy PLD plasma, a higher number of V_o_ is expected in PLD when the total pressure of the gases is low [36].

Meanwhile, ALD is a modified CVD technique that has garnered interest from both industry and academia as a thin-film deposition technique for its great step coverage and atomic-level thickness control, which are essential for designing high-aspect-ratio and three-dimensional (3D) nanoscale structures [38]. ALD typically involves precursor injection, precursor purge, reactant injection, and reactant purge, which enables the growth of a monolayer (practically sub-monolayer) with its self-saturated growth mechanism characteristic based on surface chemistry. The V_o_ concentrations are strongly influenced by deposition conditions such as the O reactant and deposition temperature, tuning the appropriate parameters is important for improving the performance of ALD-grown HfO_2_-based semiconductor devices.

**Fig. 2:**
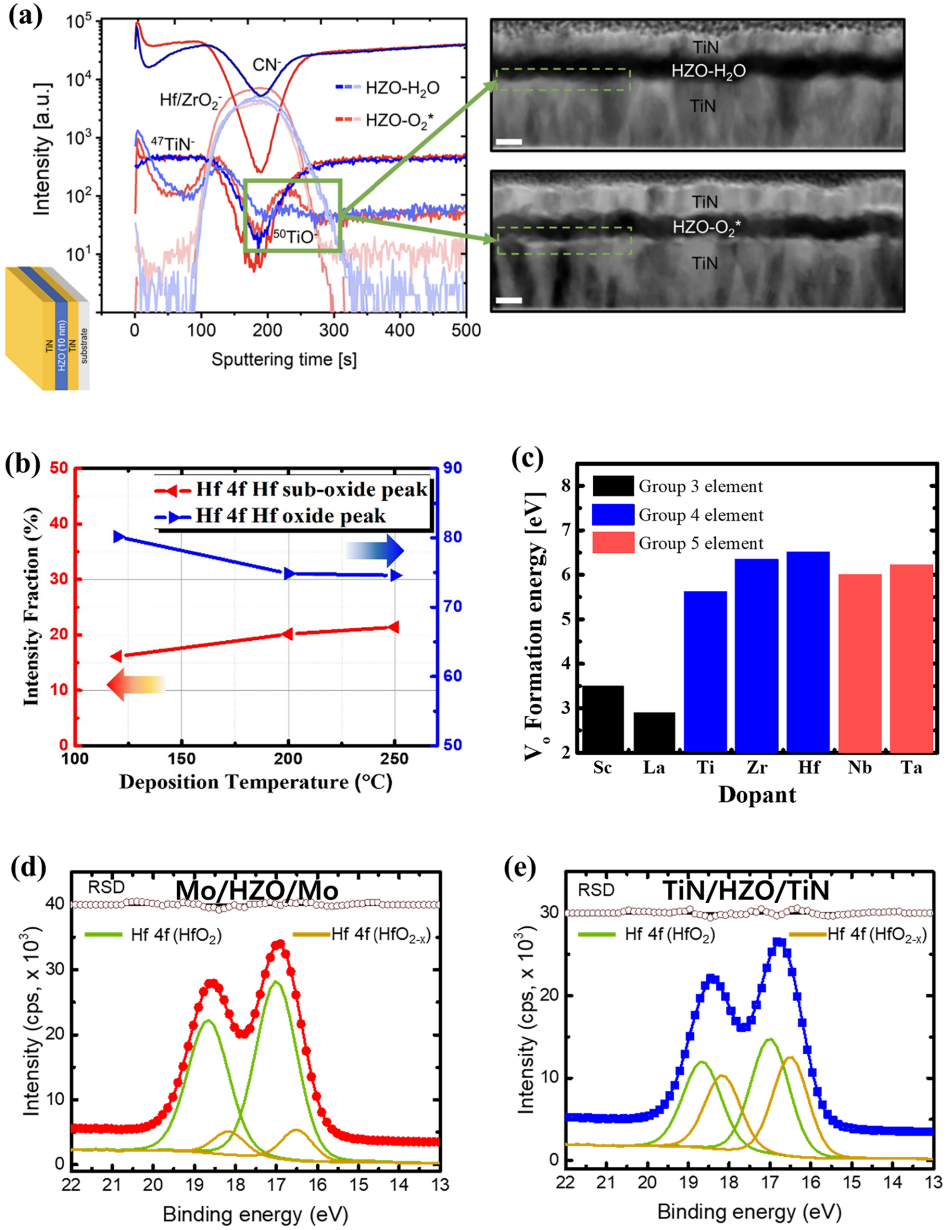
**a** Time-of-flight secondary ion mass spectroscopy (ToF–SIMS) depth profile and high-angle annular bright-field (HAABF) TEM image of TiN/Hf_0.5_Zr_0.5_O_2_/TiN capacitor using H_2_O and O_2_ plasma (O_2_^*^) reactant. **b** Intensity fraction of Hf 4f oxide (blue), sub-oxide (red) peak depending on the deposition temperature. **c** V_o_ formation energy with various dopants. Dopants are classified by chemical group. **d**, **e** XPS spectra of 2-nm Hf_0.5_Zr_0.5_O_2_ on Mo and TiN electrodes, respectively. Stoichiometric (HfO_2_) and non-stoichiometric (HfO_2–x_) peaks are deconvoluted from the Hf 4f spectrum. **a** reproduced with permission from [39]. **b** data from [42]. **c** data from [57]. **d**, **e** data from [66]

Hsain et al. studied the effect of the O reactant on the growth behavior and resulting physical and electrical properties of ferroelectric HZO film [39]. Figure 2a presents the results obtained from the time-of-flight secondary ion mass spectroscopy (ToF–SIMS) and TEM analyses by comparing the use of O_2_ plasma (O_2_^*^) and H_2_O as oxygen sources. A 10-nm HZO film was deposited on the TiN electrode and annealed at 800 °C for 30 s for film crystallization. The ToF–SIMS results revealed that films deposited by O_2_^*^ exhibited a higher ^50^TiO^−^ intensity, indicating the presence of a thicker TiO_x_ interfacial layer compared to that of the HZO film grown with H_2_O as the reactant. Typically, the interface layer is formed by scavenging O from the HZO film which increases the concentration of V_o_ within the HZO film. However, the TiN electrode is oxidized in the initial step of ALD with the highly reactive O reactant, thereby forming a TiO_x_ layer. This additional TiO_x_ layer acts as a physical barrier between the TiN electrode and the HZO film, suppressing the formation of V_o_ within the HZO film during the subsequent steps in the ALD process. Moreover, the radicals generated by O_2_^*^ enhance the reactivity of the oxygen source, resulting in a denser film with a lower concentration of V_o_.

However, plasma does not necessarily reduce the V_o_ concentration. Martínez-Puente et al. compared the HfO_2_ film properties using thermal ALD (TALD), remote plasma ALD (RPALD), and direct plasma ALD (DPALD) [40]. H_2_O and O_2_^*^ were used as O reactants for TALD and plasma ALD (RPALD and DPALD), respectively. In RPALD, the plasma source was located away from the substrate, preventing the film from directly interacting with the plasma. Conversely, in DPALD, the substrate participated in plasma generation, and the plasma was generated near the film surface. They calculated the stoichiometry of HfO_x_ films by deconvoluting the sub-oxide peak from X-ray photoelectron spectroscopy (XPS) Hf 4f and O 1s spectra, resulting in the O/Hf ratios of 1.84, 1.91, and 1.80 for TALD-, RPALD-, and DPALD-deposited films, respectively. The RPALD film exhibited a higher O/Hf ratio with an enhanced reactivity of O_2_^*^ than that for the TALD film with the H_2_O reactant. Meanwhile, the DPALD film exhibited the smallest O/Hf ratio, which can be attributed to the broken Hf–O chemical bond within the film caused by the plasma-induced damage. These facts suggest that employing a stronger O reactant such as O_2_^*^ promotes the reaction between the precursor and the reactant, resulting in a reduced V_o_ concentration in the HZO film. Nevertheless, it should be noted that the adoption of plasma does not always lead to a decreased V_o_ concentration and can damage the film.

The deposition temperature is another important factor that influences the properties of the HZO film, including the average grain size and impurity concentration, which subsequently affecting the distribution of V_o_ [41]. Choi et al. studied the effect of deposition temperature on the V_o_ concentration by deconvoluting the Hf 4f binding energy peak, which is the overlap of the HfO_2-x_ sub-oxide and stoichiometric HfO_2_ peaks, analyzed using XPS results [42]. They deposited 10-nm-thick HZO films on TiN electrodes using tetrakis (ethylmethylamino) hafnium, tetrakis (ethylmethylamino) zirconium, and H_2_O reactant by varying the deposition temperature from 120 to 250 °C. The results demonstrated that, at a deposition temperature of 120 °C, the relative areal ratio of the sub-oxide peak was 16.13%. At 250 °C, it increases to 21.42% (Fig. 2b). Similar findings were observed when deconvoluting the sub-oxide peak from O 1 s spectra, where the ratio of sub-oxide peak in O 1s increased from 2.9 to 6.15% with the increasing deposition temperature. This can be attributed to the enhanced diffusion of O from the HZO film to the TiN electrodes with an increase in deposition temperature. This phenomenon was consistently observed with various Hf precursors and reactants [43]. Kukli et al. reported the same behavior of a HfO_2_ film directly grown on an Si substrate using a tetrakis (dimethylamide) precursor and H_2_O reactant [43]. The O/Hf ratio confirmed through the time-of-flight elastic recoil detection analysis revealed that it decreased from 1.96 to 1.83 with an increase in the deposition temperature from 350 to 400 °C, indicating an increased V_o_ concentration within the film.

These results lead to the conclusion that deposition conditions such as O reactant species and deposition temperature are critical for modulating the V_o_ concentration because the formation of V_o_ is strongly correlated to the interfacial redox chemistry at the oxide/metal interfaces. This implies that the concentration and distribution of the V_o_ can be modulated by optimizing ALD conditions, which improves the physical and electrical properties of the device. Such strategic approaches are discussed in Chapters 3 and 4 for the ferroelectric and RS memory devices based on existing literature.

#### Doping

Doping is a commonly employed strategy to improve the film properties. For example, doping enhances the thermal stability and reduces the leakage currents in metal oxides [44]. In ferroelectric HfO_2_ thin films, the dopant species and concentration are the key factors that deterministically affect the polymorphism and resulting physical and electrical properties. The introduction of dopants in HfO_2_, such as Si, Al, Zr, Sr, La, Gd, or Y, has been reported to induce the formation of a metastable polar orthorhombic phase (*Pca*2_1_) at room temperature within an adequate range of dopant concentration, depending on the dopant species [45–51]. However, insufficient or excess doping can result in the formation of stable monoclinic or tetragonal/cubic phases [52]. Metal dopants can be introduced to manipulate the structural and electronic properties of HfO_2_. Several studies have revealed the close relationship between dopants and V_o_. Based on their comprehensive study of the CSD-grown doped HfO_2_ thin films, Starschich et al. [53] suggested that the radius of dopant ions is a critical factor affecting the polymorphism and ferroelectric properties. They suggested that strong ferroelectricity is expected when the dopants of which radius is larger than that of Hf. Similarly, Batra et al. reported that a clear trend was observed where dopants with a large ionic radius and low electronegativity stabilize the ferroelectric orthorhombic phase in HfO_2_ [54]. They suggested that lanthanide series elements and the lower half of the alkaline earth metals (e.g., Ca, Sr, and Ba) are favorable dopants for inducing strong ferroelectricity in HfO_2_. However, it should be noted that the dopant is not the only factor that stabilizes the polar orthorhombic phase, and therefore, many other factors (e.g., V_o_ and surface energy) should be considered to make the polar orthorhombic phase the most stable phase. Schroeder et al. [55] reported that the doping concentration range required for inducing ferroelectricity in ALD-grown HfO_2_ thin films strongly depended on the valence number and diameter of the dopants. Especially, when the valence number of dopants is different from the host cation (Hf for the case of HfO_2_), to satisfy charge neutrality, a certain amount of V_o_ must be formed.

In addition, the effect of dopants on the migration barrier of V_o_ was investigated by Zhang et al. using the density functional theory (DFT) calculations based on the generalized gradient approximation for monoclinic HfO_2_ and ZrO_2_ containing 96 atoms [56]. The migration barrier for V_o_ toward the dopant increased as the dopant radius increased, as these vacancies are unfavorable to pass through the large cations. Conversely, the migration barrier for V_o_ moving away from the dopant decreased when trivalent dopants (e.g., Al and La) were introduced due to the larger lattice relaxation compared to tetra- or penta-valent dopants, which facilitates the movement of V_o_ into a specific direction. Thus, it is straightforward that the dopant species and concentration should be strongly correlated to the V_o_ concentrations as well as their movement within the film.

Zhao et al. studied the V_o_ formation energy in HfO_2_ doped with different dopants using the Vienna ab initio simulation package (VASP) [57]. The metal dopants are divided into two groups, interstitial and substitutional according to their relative position in parent HfO_2_, by comparing the energetically more stable site of dopants within the HfO_2_ film. Dopants such as Sc, La, Ti, Zr, Nb, and Ta exhibit lower formation energies at the substitutional site, where they chemically bond with O atoms. In contrast, dopants such as Mg, Al, Ni, Cu, and Ag have lower formation energies at the interstitial sites, where they merely occupy the space between atoms. Figure 2c illustrates the formation energies of V_o_ for substitutional dopants based on the number of valence electrons. Trivalent dopants exhibit the lowest V_o_ formation energy, attributed to the instability of metal–oxygen bonds caused by a deficit of valence electrons compared to Hf atoms. However, tetra- or penta-valent dopants exhibit V_o_ formation energies similar to that of undoped-HfO_2_ because their metal–oxygen bonds are already occupied by electrons, making it difficult for these dopants to form V_o_ compared to that with trivalent dopants.

To summarize, V_o_ concentrations are strongly correlated to the valence number of dopants, because V_o_ concentration is influenced by doping of bi- or tri-valent chemical species to meet the charge neutrality condition. Another important factor in this correlation is the mobility of V_o_. Particularly, since the V_o_-dopant complex is known to be stable at lower energies, the mobility of V_o_ near bi- or tri-valent dopants should be significantly slower than that of V_o_ spatially far from the dopants. Thus, the effect of dopants on V_o_ migration and agglomeration should be studied more carefully.

#### Electrode

With the state-of-the-art dimensional scaling of memory cells, electrodes with low resistivity and large work function are considered pivotal to reducing the leakage current. Moreover, the influence of electrodes on the formation of the ferroelectric orthorhombic phase was demonstrated [58, 59]. This was attributed to the application of the mechanical in-plane stress as well as the interfacial redox chemical reactions during the fabrication processes. Consequently, beyond their electrostatic role as electrical contacts to the film, electrodes affect the concentration of V_o_ by serving as a source of O to the neighboring HfO_2_ film. In addition, the ratio of N atoms in nitride electrodes such as TiN or TaN can affect the concentration of V_o_. The N atoms can diffuse into the HfO_2_ thin film through the interface and form Hf-N bonds, causing the unwanted distribution of space charge. This can cause an asymmetric internal electric field and degrade ferroelectricity [60]. Meanwhile, more V_o_ are generated in the HfO_2_ thin film with an increase in the ratio of N atoms in TiN or TaN electrodes [61, 62]. Therefore, it is necessary to comprehend the stoichiometry of nitride electrodes and their impact on HfO_2_ thin films.

Electrodes frequently used for HfO_2_ thin films are categorized into three different groups: nitride, oxide, and metallic electrodes. TiN, a nitride electrode, has been already commercially adopted in conventional DRAM cell capacitors. However, a noteworthy concern arises in ultra-thin films, wherein an undesired TiO_x_N_y_ layer can form at the interface between the TiN electrode and the HZO film [63]. This can be attributed to the oxygen-scavenging effect of the TiN electrode, leading to oxidization of the electrode and an increase in the V_o_ concentration within the dielectric oxide film [64].

To address the reliability issues associated with the interface layer formation, extensive research has been conducted on alternative electrodes. It has been observed that conducting oxide electrodes, such as IrO_2_ and RuO_2_, reduce to Ir and Ru, respectively, at the interface between the electrode and (Hf,Zr)O_2_ [65]. Notably, Goh et al. reported that, when a RuO_2_ electrode is employed on 10-nm HZO instead of a TiN electrode, the concentration of V_o_ in the HZO film decreases. They confirmed it through XPS analysis, comparing the sub-oxide HfO_2-x_ peak intensity ratios of the two electrodes. This can be attributed to the oxide electrode supplying additional O atoms to the HZO film, resulting in a reduction of V_o_ concentration. However, Ir and Ru are less cost effective to be adopted in commercial products.

Additionally, the influence of metallic electrodes, specifically Mo and W electrodes, on the V_o_ concentration in HZO films was investigated. Figure 2d, e present the XPS analyses of 2-nm HZO films grown via ALD on Mo and TiN electrodes [66]. The non-stoichiometric HfO_2–x_ (yellow line) XPS peak was deconvoluted from the XPS Hf 4f spectrum (red line in Mo electrode, blue line in TiN electrode) to calculate the ratio of the sub-oxide peaks. The analyses revealed that the HZO grown on the Mo electrode covered a smaller portion of the HfO_2–x_ spectrum (12%) than the HZO film grown on the TiN electrode (44%). This trend persisted after rapid thermal annealing (RTA) with a decrease in the V_o_ concentration. This can be attributed to the oxidized MoO_3_ formed at the initial ALD cycles, undergoing reduction during annealing, which results in the formation of Mo and MoO_2_ species. This reduction process contributes to the decrease in V_o_ concentration within the HZO film. Consequently, the relative areal ratio corresponding to the sub-oxide peaks decreased from 12 to 3%, representing a significantly lower value than that under the pre-annealing condition. Furthermore, the impact of W electrode on the formation of V_o_ was also investigated. Yang et al. [67] employed the concept of standard formation enthalpy per oxygen of 1 mol (ΔH_f_ per O) to elucidate the reduction behavior of metal oxides. They found that the ΔH_f_ per O of WO_3_ was—280.97 kJ/mol, while HfO_2_ had a ΔH_f_ per O value of—556.6 kJ/mol. This difference suggests that WO_3_, which is formed in the initial ALD cycles, is reduced to produce W owing to its lower ΔH_f_ per O than HfO_2_.

### Migration of V_o_ and their roles in HfO_2_

In conventional MOSFET, the drift of V_o_ in high-k HfO_2_ induces hysteresis in the drain current–gate voltage transfer curve [68]. Under repeated pulses, the reversible migration of the charged V_o_ can generate the hysteresis loop, which degrades the reliability of the device. Therefore, suppressing the formation of V_o_ is desirable to enhance the performance of the electronic device.

However, the soft and reversible dielectric breakdown in the electroforming process and subsequent RS operation is associated with V_o_ migration in RRAM, which is an RS device. The resistance of the device is determined between the high resistance state (HRS) and low resistance state (LRS) under opposite voltages based on the movement of V_o_. Furthermore, the wake-up and fatigue behaviors observed in ferroelectric (Hf,Zr)O_2_ are known to originate from the V_o_ redistribution within the film (See subsection 3.2). Therefore, understanding the mechanisms and consequences of V_o_ migration is essential for comprehending the dynamics of HfO_2_ film and advancing the development of emerging devices.

In early studies, the migration of V_o_ could not be directly observed due to technological and hardware limitations. Consequently, the migration of V_o_ had to be indirectly confirmed through alternative ways. Nagata et al. utilized hard X-ray photoelectron spectroscopy (HX-PES) to observe the spectra at the interface between the Pt electrode and PLD-grown 30-nm HfO_2_ film under bias operation, providing evidence for V_o_ migration [69]. With the forward bias, the Pt–O bonding peak in O 1s spectra increased, whereas the Hf–O peak decreased, indicating the migration of V_o_ toward the electrode. In addition, Starschich et al. calculated the V_o_ migration distance during field cycling in CSD-prepared Y-doped HfO_2_ [35]. From the Mott–Gurney equation, charged V_o_ migrate approximately 6.5 nm at an electrical field of 3.25 MV/cm for 0.5 ms. While these findings provided insights into V_o_ migration, the migration process itself could not be directly captured.

However, advanced TEM techniques enable the direct imaging of light (O) and heavy (Hf) elements simultaneously. The popular high-angle annular dark-field scanning transmission electron microscopy (HAADF-STEM) collects the electrons scattered at high angles using an annular detector. This technique allows the detection of materials composed of a single atomic species or elements with similar atomic numbers. However, in the case of metal oxides with elements of different atomic numbers, detection of the scattering strength of light elements adjacent to heavy atoms is challenging. To address this issue, integrated differential phase contrast scanning transmission electron microscopy (iDPC-STEM) was introduced [70]. By combining the scattered electrons that fall inside the bright-field disk and HAADF detector, they imaged both Hf and O atoms simultaneously [71].

**Fig. 3:**
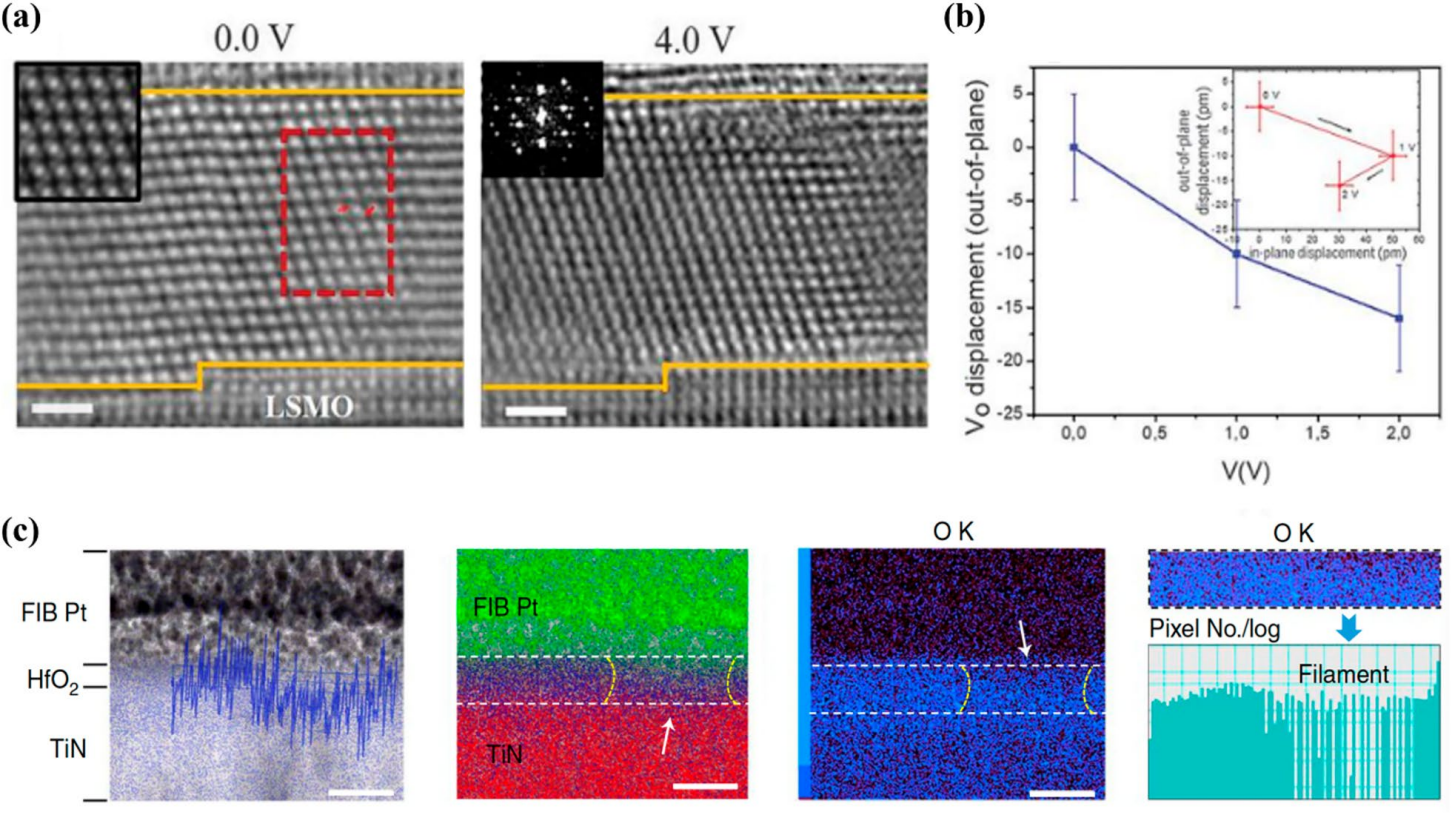
Integrated differential phase contrast scanning transmission electron microscopy (iDPC-STEM) image of rhombohedral Hf_0.5_Zr_0.5_O_2_ film under **a** 0 V (left), 4 V (right). **b** Out-of-plane displacement of V_o_ under a positive voltage. Inset shows the in-plane and out-of-plane displacements. **c** High-angle annular dark-field scanning transmission electron microscopy image overlaid with O K line profile and EDS mapping of O K edges of oxygen-deficient channel on the HfO_2_ film. **a**, **b** reproduced with permission from [71]. **c** reproduced with permission from [75]

Figure 3a illustrates the iDPC-STEM images of rhombohedral 6-nm-thick HZO epitaxially grown on an La_0.67_Sr_0.33_MnO_3_ (LSMO) electrode under 0- and 4-V bias. The two red arrows in Fig. 3a indicate two O ions chemically bonded to Hf and Zr cations. V_o_ migrates toward the bottom electrode with an increase in bias, exhibiting both vertical and in-plane migrations (Fig. 3b). Moreover, upon increasing the bias to 4 V, the epitaxial rhombohedral phase transforms into polycrystalline orthorhombic and monoclinic phases, as indicated in the inset of the fast Fourier transform (FFT) image in Fig. 3a. The stabilized rhombohedral phase under slightly O_2_-deficient condition transforms into more stoichiometric monoclinic and orthorhombic phases with oxygen supply from the bottom LSMO electrode. However, the rhombohedral phase is recovered under − 4 V through the reversible migration of O atoms (vacancies). In addition, by employing a noble electrode (Au), the study discovered that the HZO film itself acted as a V_o_ supplier, resulting in a phase transition between the rhombohedral and monoclinic phases. These observations suggest that the migration of V_o_ can induce phase transitions in epitaxial rhombohedral HZO and are dependent on the ferroelectricity in the HZO film.

In RS devices, the electric field-dependent migration of V_o_ plays a crucial role in the entire switching process. In the V_o_-mediated filamentary switching type, the switching mechanism involves the formation of a V_o_ CF, which results from the localized migration of V_o_ when voltage is applied. The forming process is required to initialize the formation of CFs, and this initialization is triggered by the electric field-driven dielectric breakdown process. During the forming process, oxygen ions dissociate from the HfO_2_ matrix and migrate from the cathode toward the anode under an external electric field, which initializes the nucleation and growth of V_o_ CFs in the HfO_2_ layer. Under subsequential operation (RESET and SET) biases, the V_o_ migrate, and the migration is driven by voltage. This phenomenon triggers repeated CF rupture, connection, and operating OFF/ON in the RS devices. The accurate observation and understanding of V_o_ dynamics are important for implementing controllable RS operations because these electroforming and subsequent RS operations are associated with V_o_ migration.

Unlike the highly mobile Ag or Cu in electrochemical metallization cell-type devices, the motion of oxygen in RS devices cannot be observed easily as it is a light element and suffers from ambient occlusion. Therefore, studies used TEM for observing the chemical composition and morphology of the device to directly identify V_o_ migration. In these studies, the change in the chemical composition in the V_o_ region was studied by EELS or high-resolution energy dispersive X-ray spectroscopy (EDS). The EELS is an appropriate method for light element detection because its contrast is not proportional to the atomic number but related to the inelastic interactions of the element with the materials. Therefore, EELS analysis can be used to observe changes in the oxygen concentration after RS operation with a low-energy-loss spectrum. Prior studies exploited EELS with STEM to observe electrical stress-mediated V_o_ migration in HfO_2_-based RS devices (Fig. 3c) [72, 73]. Based on the local chemical composition and phase, the STEM-EELS chemical maps of the V_o_ migration region can be recorded with high energy (1.6 eV) and spatial resolution (0.5 nm), which is evidence for the localization regions of the individual V_o_ in HfO_2_.

Notably, the detailed characteristics, which include atomic structure and phase transformation in and near the V_o_ migration region, were demonstrated through high-resolution transmission electron microscopy (HR-TEM) observations [74]. Yin et al. studied crystallite kinetics coupled with V_o_ migration using TEM in a W/HfO_y_/HfO_x_/Pt stacked structure. After the formation process, they discovered directionally aligned crystalline regions consisting of monoclinic and orthorhombic oxygen-deficiency phases in the initial amorphous HfO_2_. Their TEM observations demonstrated that the extrusion of V_o_ migration resulted in structural modifications involving crystallite separation, phase transformation, and misalignment. Further, Yang et al. attributed the difficulty in the direct visualization of oxygen ion motion to the commonly used electron microscope imaging focusing on the mass properties of ions [75]. Therefore, they combined TEM with in situ electrostatic force microscopy (EFM), which exploited both the mass and charge attribution of oxygen ions. Both accumulation and V_o_ migration can be detected by the electrostatic force between the probe and the sample because EFM is sensitive to charge accumulation. Moreover, the formation of conduction channels within the HfO_2_ layer was directly detected by high-resolution STEM and EDS analyses. Their results can be utilized for probing the ion-transport dynamics in solid electrolytes and for understanding the switching mechanism of RS devices.

Previous studies indirectly confirmed V_o_ migration through theoretical computations and alternative methods such as spectroscopy. However, recent advances in TEM techniques, specifically iDPC-STEM, enabled the direct imaging of V_o_ migration. In RS devices, accurately observing and understanding V_o_ dynamics is essential because electroforming and the subsequent RS operations rely on V_o_ migration. Observation techniques such as EELS, HRTEM, and in situ EFM were employed to study V_o_ migration and its effects on the behavior of the device, and the results provided valuable information on the chemical composition, phase transformation, and structural modifications caused by the migration.

### Aggregation of V_o_ in HfO_2_

**Fig. 4:**
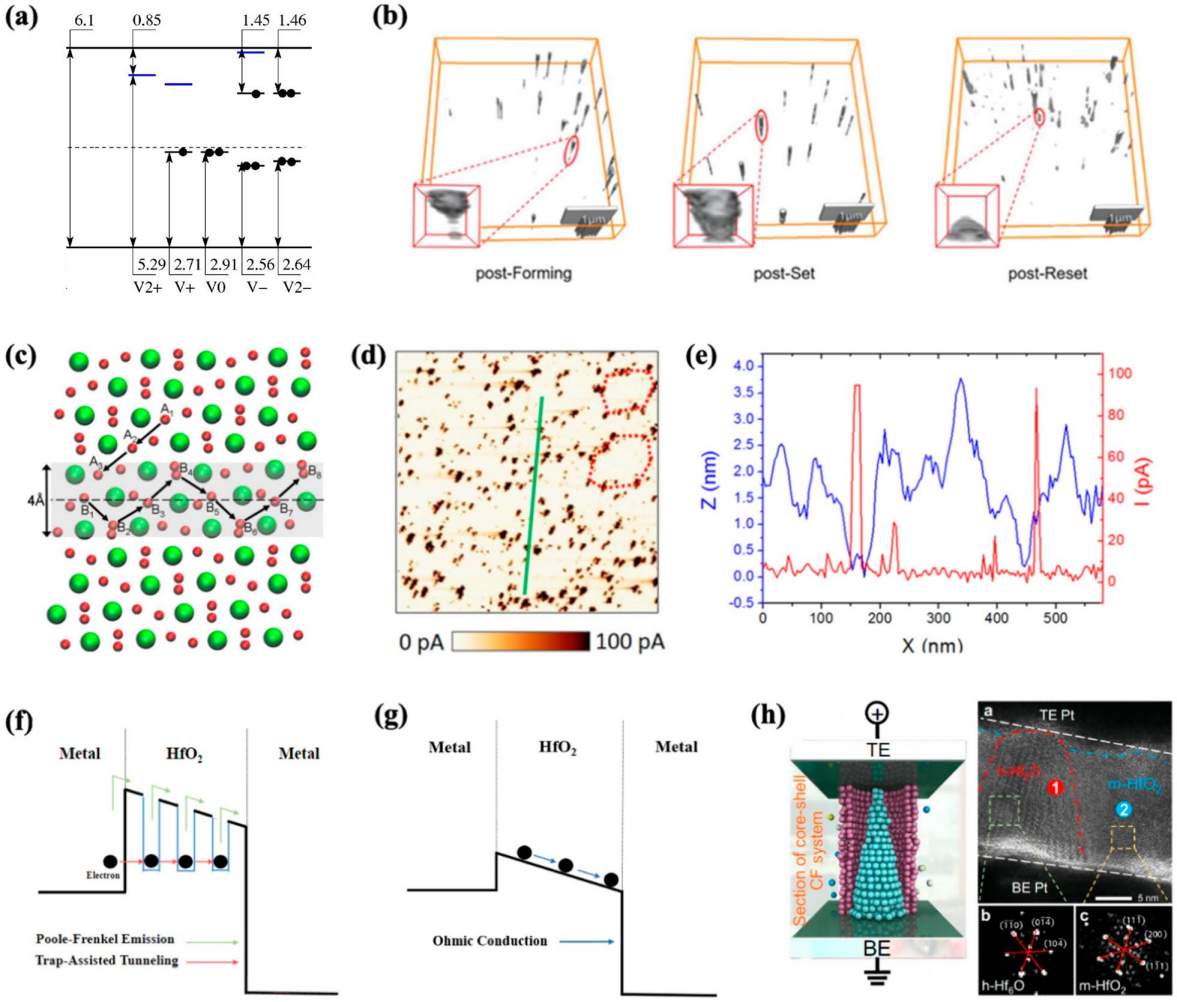
**a** Bandgap of HfO_2_ with defect states depending on the charge state of V_o_. **b** Reconstructed 3D C-AFM images of V_o_ evolution and migration in HfO_x_-based RS device.** c** Atomic structure of monoclinic HfO_2_ with GB at the center (dashed line). The 4-Å-wide shadow region near the GB is a favorable region for V_o_ aggregation.** d** Current scan map using C-AFM of polycrystalline HfO_2_. **e** Topography (blue) and current (red) data along the green solid line in** d**. Schematic of conduction mechanism in HfO_2_ **f** before and **g** after breakdown. **h** Schematic and HRTEM of a complete CF resulting from V_o_ aggregation in an LRS device with a typical polymorphous HfO_x_ region, namely h-Hf_6_O and m-HfO_2_ regions **a** reproduced with permission from [76]. **b** reproduced with permission from [80]. **c** reproduced with permission from [83]. **d**, **e** reproduced with permission from [184]. **h** reproduced with permission from [79]

Understanding the aggregation behavior of V_o_ is crucial for comprehending the conduction mechanism because the formation of V_o_ chains can significantly impact the conductivity of the film. Figure 4a depicts the distribution of defect levels within the bandgap of a monoclinic-phase HfO_2_ film concerning the charge states of V_o_ at their most favorable sites: positively charged V_o_^+2^, V_o_^+1^ threefold coordinated sites, and neutral and negatively charged V_o_^0^, V_o_^−1^, and V_o_^−2^ fourfold coordinated sites [76]. Gavartin et al. employed the B3LYP hybrid density functional electronic structure calculations on monoclinic-phase HfO_2_ with 96 atoms. The results indicate that V_o_^+2^ creates one empty state 0.85 eV below the conduction band (CB), while V_o_^−2^ generates two fully occupied states—one 1.46 eV below the CB and the other near the intrinsic Fermi level. For V_o_^+1^ and V_o_^−1^, they become magnetic defects, occupying half of the defect states. In case of a V_o_^0^, it generates fully occupied state near intrinsic Fermi level. These findings suggest that V_o_ serves as an electron- and hole-localization center and a medium for trapping and transporting carriers. Consequently, the aggregation of V_o_ generates conductive paths or preferential sites for charge accumulation, causing film degradation or breakdown. The aggregation of V_o_ can occur through two distinct mechanisms: the clustering of pre-existing V_o_ or the creation of new V_o_ near the pre-existing ones under a strong electric field (1–2 MV/cm), weakening the Hf–O bond and promoting the generation of new V_o_ and O interstitials.

Gao et al. conducted a DFT-based calculation to investigate V_o_ aggregation in amorphous HfO_2_ by employing 9 periodic models, containing 324 atoms each. They discovered that the V_o_ formation energy adjacent to the pre-existing V_o_ was lower than the formation energy of a single V_o_ [77]. This energy decreased with an increase in the number of adjacent V_o_ implying that the rate of vacancy formation increased significantly once V_o_ is generated. This can be attributed to distortion from the surrounding system, which facilitates the generation of new V_o_. Furthermore, the aggregation of V_o_ amid electron injection was examined [78]. Injection of electrons from the electrode is inevitable under the high-electric-field condition which causes thermal fluctuations in O ions. It dislodges O atoms from their original sites and causes the formation of V_o_–O interstitial pairs. The study also determined that the production of a V_o_–O interstitial pair required energy of 1.19 eV from the perfect cell. This energy cost reduces to 0.96 eV near V_o_. Consequently, V_o_ are more likely to form near the existing ones, favoring aggregation rather than distributing uniformly throughout the film.

Extensive nanoscale physicochemical analyses have been conducted to directly observe nanoscale V_o_ aggregation [79–82]. However, despite its importance, observation of V_o_ aggregation is challenging due to the small size of the filaments and minimal compositional difference from the surrounding matrix material. Among the various tools used for observing V_o_ aggregation, conductive atomic force microscopy (c-AFM) is widely employed because it can locally measure the electrical properties of sample surfaces with high spatial resolution. Celano et al. directly observed the morphology of a single CF during the forming process in a scaled Ru/Hf/HfO_2_/TiN device using a technique [81] that integrates AFM-based tomography (scalpel-SPM) with the high lateral resolution of c-AFM and sub-nanometer vertical resolution through a controlled removal procedure. This slice-and-view tomography technique provided a 3D characterization of a single CF, demonstrating its size (< 10 nm) and constriction regions determined by V_o_ aggregation under an electric field. According to their observations, the CF exhibited a conical shape located at the interface of the oxide-inert electrode, and this morphology correlated with the electrical conditions. Their results elucidated the morphological features of CF resulting from V_o_ aggregation under specific electric field conditions.

Although the study demonstrated the aggregation of V_o_ in a single CF during the forming process, it does not cover all the RS states resulting from various operations, such as forming, SET, and RESET. Wei et al. analyze CFs in the post-forming, post-SET, and post-RESET states in a Pt/HfO_x_/TaO_y_/TiN device using c-AFM techniques with 3D reconstruction (Fig. 4b) to comprehensively analyze CF changes in all three resistance states, including the quantity, morphology, and rupture location in the high-resistance state [80]. They acquired the reconstructed 3D images of CFs by combining the two-dimensional (2D) conductivity profiles collected from each exposed surface of the top electrode (TE) and switching layer, removed by a conductive diamond tip at different heights. Furthermore, multiple conductive channels in each resistance state could be observed because the 2D conductivity profiles cover the entire device. The CFs after operation under each state exhibited typical morphologies, including hourglass, inverted-cone, and short-cone, respectively. During the forming process, a positive voltage was applied to the bottom electrode (BE), causing oxygen ions to dissociate from the HfO_x_ layer and migrate toward the BE, while leaving V_o_ in the HfO_x_ layer. Positive feedback from the electric field accelerates oxygen ion migration toward the oxygen reservoir layer (oxygen-deficient TaO_y_ layer in the bilayer structure HfO_x_/TaO_y_), which results in V_o_ aggregation, thereby forming tapered CFs. The mechanism of V_o_ migration during the SET operation is similar to the forming process; however, thicker CFs are observed after the operation. Subsequently, during the RESET process, an opposite external electric field is applied, causing oxygen ions to migrate out of the oxygen reservoir layer and upward to merge with V_o_, which results in the rupturing of V_o_ CFs from the upper end near the TE and switching of the device from LRS to HRS. Subsequently, this leads to the formation of broken/newly generated cone-shaped filaments at the HfO_x_/oxygen reservoir layer interface.

Zhang et al. employed HRTEM to comprehensively investigate the crystal structure of a CF resulting from V_o_ aggregation, and they revealed that the V_o_ CF possesses the core–shell structure (oxygen-deficient CF and the corresponding oxygen-rich shell) consisting of metallic hexagonal-Hf_6_O (h-Hf_6_O) and its crystalline surroundings (monoclinic and tetragonal HfO_x_) [79]. Based on their theoretical and experimental investigation, they reported that the RS process of HfO_2_-based devices involve a phase transition of the CF shell from the monoclinic to the tetragonal phase. This transition occurs in the CFs surrounding both the complete and ruptured h-Hf_6_O. This core–shell structure was observed by another study while analyzing a HfO_x_-based RS device using synchrotron-based scanning transmission X-ray microscopy (STXM) [82]. The STXM system is employed for the nondestructive observation of the in situ switching of HfO_2_-based RS devices and the analysis of the variations in the chemical composition and positions associated with V_o_ aggregation. After completing the ON/OFF switching cycles, they examined a cell by capturing images at various X-ray energies specifically tailored to the O K-edge (570 eV). The images revealed a localized region consisting of a darker ring (low-conductivity, oxygen-rich region) surrounding a brighter center (high-conductivity, oxygen-deficient region).

Notably, V_o_ aggregation occurs in specific regions rather than arbitrary locations. Figure 4c shows the atomic structure of monoclinic HfO_2_ with the dashed line in the middle indicating the grain boundary (GB) [83]. Mckenna et al. employed nudged elastic band (NEB) calculations and the VSAP code to comprehensively characterize the behavior of V_o_ across the film. They observed a reduction of diffusion energy barrier approximately 0.7 eV near the GB compared to the bulk, indicating increased stability of this region with a lower formation energy compared to other regions [84, 85]. They attributed the cause of this phenomenon to the variations in bond length and electrostatic potential near the GB, which in turn enhanced the relaxation of ions.

Another study employed c-AFM to experimentally verify the aggregation of V_o_. Figure 4d illustrates the CAFM current scan map of polycrystalline HfO_2_ using a conductive tip as the TE. Stronger currents were observed in specific regions, which corresponded to areas with lower topographical heights. These depressions were produced by thermal grooving, which is frequently observed at GBs [86, 87]. The difference in free energy between the GB and the surface of the film results in the formation of a topographical groove with a certain angle within the polycrystalline film. Therefore, the observed strong current near the GB can be attributed to the increased tunneling current and aggregation of V_o_.

Finally, Fig. 4f illustrates the conduction mechanisms responsible for the leakage current produced in the HfO_2_ film. Extensive studies have worked on these mechanisms, including the Poole–Frenkel (PF) emission, Schottky emission, trap-assisted tunneling (TAT), Fowler–Nordheim (FN) tunneling, and space charge limited current [20, 88–90]. However, the precise mechanism could not be explained without considering the contribution of traps. The Schottky emission and FN tunneling model contain abnormal parameters and fail to generate an accurate interpretation of the experimental results, which demonstrate an increase in leakage current with higher V_o_ concentrations [20]. Trapped electrons can transition from their localized state using two mechanisms: thermal fluctuation and tunneling. In PF emission, thermal fluctuations provide sufficient energy for electrons to migrate to the CB and relax into a different localized state. Notably, under strong electric fields, electron hopping can be facilitated without significant thermal fluctuations [91]. Meanwhile, the TAT model describes the electron tunneling phenomenon between traps providing additional tunneling paths that increase the probability of electrons tunneling through the barriers [92]. These observations demonstrate the relationship between V_o_ concentration and leakage current, i.e., a high V_o_ concentration leads to a high leakage current. However, the repetitive generation of V_o_ under a high electric field and electron injection form conductive pathways across the film, ultimately resulting in a hard breakdown (Fig. 4g). In summary, aggregated V_o_ near the GB serve as a charge-transportation medium, while excessive V_o_ concentration can cause the hard breakdown of the film.

To summarize, the aggregation behavior of V_o_ in HfO_2_ films plays a crucial role in their conductivity and conduction mechanisms. V_o_ chains significantly influence the film’s conductivity by acting as carrier traps and transport centers. Several studies have utilized computational methods to investigate the behavior of V_o_ and revealed the charge states of V_o_ create different degrees of defect within a bandgap. V_o_ aggregates through two mechanisms: clustering of pre-existing V_o_ and generation of new V_o_ near existing ones under strong electric fields. Experimental techniques like c-AFM and HRTEM were employed to observe V_o_ aggregation at the nanoscale. The formation of V_o_ chains and their morphological features, including hourglass, inverted-cone, and short-cone, have been directly observed. The crystal structure of V_o_ chains is a core shell with h-Hf_6_O and its crystalline surroundings (Fig. 4h). Particularly, V_o_ aggregation preferentially occurs at GBs owing to reduced diffusion barriers and lower formation energies. Moreover, the aggregation of V_o_ near GBs produces higher tunneling currents and increased conductivity. Leakage currents in HfO_2_ films are influenced by the V_o_ concentration, i.e., high concentrations result in increased leakage. However, excessive V_o_ generation under high electric fields and electron injection has two outcomes: a soft breakdown, which induces the formation of CFs for RS behavior, or a hard dielectric breakdown of the film. Therefore, understanding V_o_ aggregation will help comprehend the conduction mechanisms, degradation processes, and RS behavior in HfO_2_ films, as it impacts their conductivity and leads to the formation of CF or film breakdown.

## Effects of V_o_ on electrical properties reliability of ferroelectric HfO_2_

The spatial distribution and average concentration of V_o_ within HfO_2_ thin films significantly influence their polymorphism and the resulting ferroelectric properties, as well as reliability metrics such as switching endurance that are the major performance parameters of semiconductor devices based on HfO_2_-based ferroelectrics [29, 37, 93]. Studies reported that the concentration of V_o_ influences the relative free energy of the metastable crystallographic phase, including the ferroelectric orthorhombic phase [23, 94]. Interestingly, their relative location could also influence the polymorphism of HfO_2_-based thin films [23]. Moreover, charged V_o_ could be drifted by repeated electrical field cycling, causing wake-up, fatigue, and hard breakdown, which are typically observed during the endurance tests of ferroelectric HfO_2_-based capacitors [35, 95, 96]. Therefore, understanding the effect of V_o_ concentration and spatial distribution on the ferroelectric properties and reliability of HfO_2_ devices is important for advancing HfO_2_-based ferroelectric memory devices.

The effect of V_o_ in HfO_2_-based RS memories is even more comprehensible owing to the pivotal role played by filament formation and rupture resulting from V_o_ accumulation and electrically or thermally driven migration in driving the resistive change. Precise control over V_o_ is crucial for guarantee the reliable memory-switching behavior of HfO_2_-based RS devices. Therefore, the possible factors that can influence V_o_-mediated RS behavior must be identified, and reliable methods to control V_o_ dynamics must be established.

Therefore, in this section, the effects of location, concentration, state of charge of V_o_ on the phase stabilization of HfO_2_, and migration of V_o_ induced by electrical field cycling are comprehensively reviewed and critically discussed based on extant literature. Furthermore, we also provide some perspectives on strategies to improve the performances and reliability of semiconductor devices based on ferroelectric HfO_2_ by controlling the V_o_ concentration based on the current understanding of them.

### Effect of V_o_ on ferroelectric properties of HfO_2_

**Fig. 5:**
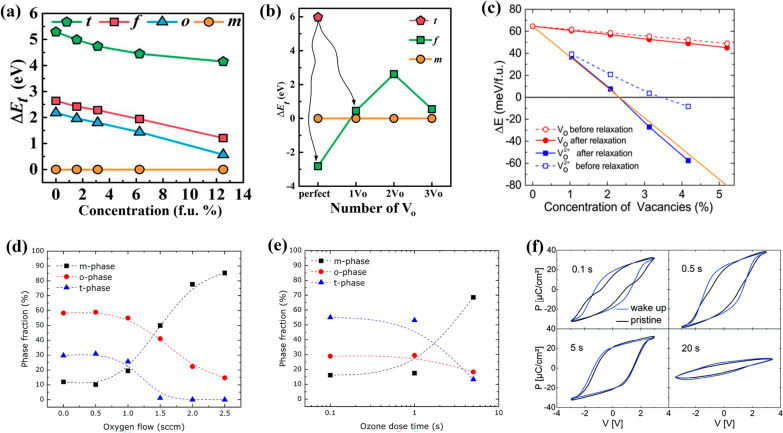
Total energies of the various crystal phases of HfO_2_ with different V_o_ concentrations of the **a** bulk and **b** interface region. **c** Energies of the orthorhombic *Pca*2_1_ phase of HfO_2_ with different V_o_ states of charge and relaxation conditions relative to the monoclinic phase (ΔE) as a function of V_o_ concentration. Phase content of 10-nm HZO samples as a function of the **d** ozone dose time during the ALD process and **e** oxygen flow during the sputtering. The phase portions are extracted and calculated by the GIXRD pattern. **f** Polarization–electric field curves of the 10-nm samples vs. ozone dose time during ALD process. **a**, **b** reproduced with permission from [23]. **c** reproduced with permission from [94]. **d**, **e** reproduced with permission from [227]. **f** reproduced with permission from [37]

Zhou et al. investigated the effect of V_o_ on the crystal phase of HfO_2_ using the first-principles method based on DFT calculations. Figure 5a illustrates the total energy of the various crystal phases of HfO_2_ in the bulk regions of the HfO_2_ thin film as a function of V_o_ concentration [23]. Here, t, f, o, and m refer to the tetragonal *P*4_2_/*nmc*, polar orthorhombic *Pca*2_1_, nonpolar orthorhombic *Pbca*, and monoclinic *P*2_1_/*c* phases, respectively. For the bulk HfO_2_ examined in Fig. 5a, the monoclinic phase is the most stable phase with the lowest total energy when the V_o_ concentration is zero. According to the phase diagrams of HfO_2_ and ZrO_2_, the relative free energies of the tetragonal and cubic phases decrease with increasing temperature. Consequently, the thermodynamically stable phase sequentially changes from the monoclinic phase to the tetragonal and cubic phases at 1,943 and 2,573 K in HfO_2_ and at 1,473 and 2,743 K in ZrO_2_, respectively [5, 97].

However, as the V_o_ concentration increases, the differences in total energy between the monoclinic and other metastable phases, including tetragonal and polar orthorhombic phases, decrease. As the crystallographic origin of ferroelectricity in polycrystalline HfO_2_ thin films is the formation of the polar orthorhombic phase [10, 52, 98], an adequate range of V_o_ concentration in the films would be beneficial for inducing ferroelectricity. According to previous studies, in epitaxial doped (Hf,Zr)O_2_-based films, the crystallographic origin of ferroelectricity is the formation of the rhombohedral phase (space group: *R*3 or *R*3*m*). However, in this study, polycrystalline films are the focus. Notably, several aspects related to the effect of V_o_ on polymorphism and the resulting ferroelectricity calculated from the computational simulation should be carefully considered. First, the maximum V_o_ concentration that does not crystallographically distort the original crystal structure should be considered, similar with the effect of chemical doping. Batra et al. [54] examined via high throughput calculations the effect of doping various metallic cations into HfO_2_ thin films. They showed that an excessive dopant concentration severely distorted the crystallographic structure by breaking the original symmetry in the parent phase. Similar effects are also expected with a high V_o_ concentration, which should be considered. Moreover, excessive V_o_ concentration could be the reason for the high leakage current produced by the formation of localized energy levels in HfO_2_ thin films, which would be another limiting factor of V_o_ for inducing ferroelectricity in HfO_2_. Second, an excessive V_o_ concentration could destabilize the polar orthorhombic phase because the total energy of the tetragonal phase tends to more rapidly decrease with increasing V_o_ concentration [50]. Third, most computational studies examined the total energy at 0 K, so the effect of entropy-related terms should also be considered. Particularly, high-temperature phases such as the tetragonal and cubic phases have entropy values significantly higher than those of the polar orthorhombic and monoclinic phases, which was proven by the energy crossover observed in previous studies when an entropy term was added to Gibbs free energy [6].

Despite these limitations, the effect of V_o_ on the relative free energy of various polymorphs is scientifically meaningful and consistent with experimental observations. The effect of V_o_ concentration would thermodynamically and/or kinetically alter the final polymorphs formed in HfO_2_-based thin films. The free energy difference between the polar orthorhombic and stable monoclinic phases is, in fact, excessively large to be overcome solely by the V_o_ effect. However, its synergy with various factors, such as surface energy, doping, and stress, could cause the formation of the polar orthorhombic phase. According to the kinetic model, the precursor tetragonal phase formed at elevated temperatures is a key core process, which might be facilitated by the effect of V_o_. The subsequent competition between the tetragonal and polar orthorhombic phases should also be strongly correlated with this effect. Therefore, for both models, the V_o_ should be a critical factor influencing polymorphism and the resulting ferroelectricity [99–101].

In addition to the concentration of V_o_, spatial distribution is another critical factor determining the ferroelectricity of HfO_2_. Zhou et al. examined the effect of V_o_ on the total energy of various polymorphs of HfO_2_ and revealed that the free energy values of the polymorphs depended on the location of V_o_. Figure 5b illustrates the total energy of the tetragonal, polar orthorhombic, and monoclinic phases as a function of the number of V_o_ at the interface through DFT calculation was with a 1 × 1 × 4 supercell, where the total number of Hf and O atoms were 32 and 16, respectively. In the absence of V_o_ (perfect) in the interfacial region, the magnitude of reduction in free energy of the polar orthorhombic phase could further widen, resulting in a greater stabilization of the phase. However, with increasing number of V_o_ beyond 1, the monoclinic phase becomes the most stable phase. Here, 1, 2, and 3 V_o_ refer to the 1, 2, and 3 V_o_ relative to the total oxygen position in the supercell. This led us to interpret that the ferroelectricity within this phase can be stabilized by maintaining a low concentration of V_o_ at the interface of the thin film, unlike at the bulk region.

The V_o_ in oxide materials such as HfO_2_ exist in a neutral or positive-charge state [102, 103]. Therefore, the effect of the state of charge of V_o_ on the relative free energy of various polymorphs of HfO_2_ must be investigated. Figure 5c illustrates the difference in total energy between the polar orthorhombic and monoclinic phases of HfO_2_ due to the concentration of V_o_ with neutral or positive states of charge reported by He et al. [94]. They calculated the total energy using the DFT method. To examine the effect of the state of charge of V_o_ on phase stability, they defined ΔE as the difference in energy between the orthorhombic *Pca*2_1_ phase and monoclinic *P*2_1_/c phases and a function of the concentration of V_o_. The orthorhombic *Pca*2_1_ phase and monoclinic *P*2_1_/c phases are modeled with 2 × 3 × 2 supercells with 96 O atoms. Here, removal of one oxygen atom is expressed at HfO_2–x_, where x = 2.08%. In addition, to understand the effect of atomic relaxation, the scenarios of before (dash) and after (solid) lattice relaxation are compared in Fig. 5c. The red line in Fig. 5c indicates that, even as the concentration of neutral V_o_ increases and the energy difference decreases, the polar orthorhombic phase continues to possess a higher total energy than the monoclinic phase. Conversely, the blue line, which corresponds to the change in energy of the polar orthorhombic phase induced by Vo^2+^ with a positive state of charge, indicates that, for the trend, the energy difference between the polar orthorhombic and monoclinic phases decreased significantly. In addition, an energy crossover was observed at approximately 2.2% and 3.1% for the relaxed and unrelaxed lattices, respectively. Hence, positively charged V_o_^2+^ has a markedly stronger effect than neutral V_o_. Additionally, a greater stabilization of the polar orthorhombic phase can be observed at lower V_o_ concentrations under the relaxed state of the lattice compared to that under the unrelaxed state.

Furthermore, He et al. [94] reported that a positively charged V_o_^2+^ had a lower diffusion activation energy than a neutral V_o_. The Vo diffusion activation energy of the positive and neutral V_o_, calculated through DFT calculations, were 2.49–2.89 and 0.89–0.98 eV, respectively, which are consistent with the values reported in previous studies [104–107]. He et al. explained that in case of a neutral Vo, localized electrons within the HfO_2_ band gap interfere with the migration of the V_o_. In contrast, for positive V_o_, the bandgap does not contain effective charge, which results in its significantly lower V_o_ diffusion activation energy. This phenomenon was also observed in ZrO_2_ [108, 109] and other oxides [110, 111].

In summary, a high concentration of charged V_o_ could reduce the total energy difference between the polar orthorhombic phase and the monoclinic phase. Additionally, positively charged V_o_^2+^ have a lower diffusion activation energy than neutral V_o_, which could explain the V_o_ migration experimentally observed in different studies. The effect of oxygen vacancies on the ferroelectric properties of the HfO_2_ film requires should be investigated further. Jaszewski et al. conducted XPS and positron annihilation spectroscopy analyses for V_o_ characterization of 20 nm HfO_2_ deposited by magnetron sputtering [30]. They confirmed that the concentration of neutral V_o_ increased after the heat treatment of the thin film. However, ferroelectricity with a maximum of 2P_r_ of about 17.4 μC/cm^2^ appeared in the HfO_2_ thin film, suggesting that neutral V_o_ can also have an important influence on the ferroelectric properties of HfO_2_. V_o_, which is commonly discussed in HfO_2_ ferroelectric applications, is a positively charged defect, and therefore, the positively charged V_o_ is discussed in the subsequent sections.

Ferroelectric HfO_2_ thin films reported in various previous studies were typically deposited via the ALD process [112–116]. As discussed in Sect. 2.1, the ALD process parameters, such as the O source or deposition temperature, distinctively alter the concentration of V_o_ in HfO_2_ thin films. Furthermore, as the concentration and migration of V_o_ can impact the crystal phase of the thin film, the oxygen source density or/and injection time during the ALD process can have a pronounced effect on the ferroelectric properties [117, 118].

Mitmann et al. investigated the ferroelectric properties of a HZO thin film by varying the ozone dose time during ALD process [37]. Figures 5d, e display the evolution of phase portion of TiN/HZO/TiN capacitors influenced by the oxygen flow or ozone dose time in the sputtering or ALD process, respectively. Figure 5f shows the polarization–voltage curves of the TiN/HZO/TiN capacitor deposited via the ALD process. Here, the relative phase fractions were extracted from grazing incidence X-ray diffraction (GIXRD) measurement results. Oxygen flow is the flow rate of injected oxygen gas during the sputtering process, and ozone dose time is the time taken to inject ozone flow during the ALD process. Although precise quantification may be difficult, it is reasonable that the concentration of V_o_ in the HZO thin film might decrease due to the increase in the oxygen flow and ozone dose time. For the HZO thin film deposited via the sputtering process, an increase in the monoclinic phase fraction and a decrease in the orthorhombic phase were observed with an increase in the oxygen flow. In case of the HZO thin film deposited by the ALD process, similarly, Similarly, as the ozone dose time increased, the monoclinic phase fraction increased, and the orthorhombic phase fraction decreased. The similar trend was observed by Pal et al. where the fraction of the monoclinic phase increased and leakage current decreased with an increase in the ozone dose time during ALD [119]. This behavior may be explained by the significant decrease in the concentration of V_o_ in the thin film due to excessive injection of oxygen sources. Additionally, this result alludes to the following tendencies: (1) An increase in the concentration of V_o_ in the HZO thin film may further stabilize the monoclinic phase; and (2) excessively high or low V_o_ concentrations may rather make the orthorhombic phase unstable. Therefore, an oxygen dosage optimized for the V_o_ concentration and enhancing the ferroelectric properties should be supplied to the HZO thin films.

In the polarization–voltage curve of Fig. 5e, the HZO thin film yields the maximum double remanent polarization (2P_r_) value of approximately 42 μC/cm^2^ for an ozone dose time of 0.5 s. In addition, a distinctly low P_r_ value is observed in films with the shortest and longest ozone doses of 0.1 and 20 s, respectively. The notable point is the difference between before (black line, pristine) and after (blue line, wake up) the wake-up cycle of HZO in Fig. 5e. The ferroelectric HfO_2_ thin films show phenomenon in which P_r_ value increases by repeated electric-field cycling, so-called the wake-up effect [120–124]. The origin of this effect is still debatable, but the general consensus is that it is the redistribution of concentrated V_o_ in the interface region and transition from the tetragonal to orthorhombic phases [95, 100, 125]. Based on this, the wake-up effect according to the phase fraction can be identified by the polarization–voltage curve in Fig. 5e. The HZO thin film with an ozone dose time of 0.1 s exhibits a pinched hysteresis loop with a relatively low P_r_ value in the pristine state. After the wake-up cycle, however, distinct changes, such as an increase in P_r_ and an open hysteresis loop, are observed. On the other hand, in the HZO thin film with an ozone dose time of more than 5 s, where the tetragonal phase fraction is remarkably reduced, no evidence of the evolution of P_r_ value and/or hysteresis loop was found during the wake-up field cycling. This result could be understood through the decrease in the tetragonal phase fraction in the pristine state, one of the hypothetical origins of the wake-up effect [100, 123, 124]. The decreased tetragonal phase fraction can be explained from a previous report that the relative fraction of the tetragonal phase may increase as the V_o_ concentration in the ferroelectric HfO_2_ thin film is increased [50]. It could be the reason for the strongest wake-up effect in the HZO thin film, which suffered the shortest ozone dose time, shows the highest V_o_ concentration. Furthermore, if the ozone dose time is increased, the injection of more oxygen atoms can decrease the relative fraction of the t-phase. This can lead to a high P_r_ value and relaxation of the wake-up effect. The electrical properties, including the wake-up effect, of the HfO_2_ thin film under electric-field cycling will be expounded in Sect. 3.2. Notably, The effect of V_o_ can appear as the formation of a local electric field or domain-wall pinning, which is different from the effect on the relative free energy of the various polymorphs discussed earlier. However, with any intermediate mechanism, the high V_o_ concentration in the pristine state would cause a rather strongly pinched hysteresis owing to the wake-up effect.

A phase change from tetragonal to the orthorhombic phase is considered the origin of the wake-up effect of ferroelectric HfO_2_, as stated previously. However, a recent study reported that the wake-up effect and fatigue phenomenon could be attributed to the phase transition from the antiferroelectric orthorhombic *Pbca* phase to the ferroelectric orthorhombic *Pca*2_1_ phase [9]. Cheng et al. reported the movement of the oxygen atom and the phase transition mechanism according to the electric field cycling of the HZO thin film. The STEM-annular bright-field (ABF) mode, which has a high detection sensitivity for oxygen ions, was used for the analysis. The coexistence of *Pbca* and *Pca*2_1_ phases is confirmed in the pristine state of HZO film; however, the *Pca*2_1_ phase becomes more dominant than the *Pbca* phase in the wake-up state. From the STEM-HAADF analysis, the thickness of tetragonal phase increased from 0.75–1.0 nm to 0.94–2.11 nm after electric field cycling. This result contrasts the common theory that the HZO thin film suffers phase transition from the tetragonal phase to the orthorhombic phase after electric field cycling. The increase in the tetragonal phase fraction after electric field cycling can be attributed to an increase in the concentration of V_o_, which stabilizes the tetragonal phase at the interface between the HZO thin film and the TiN electrode. This result is a significantly different interpretation from the wake-up and fatigue mechanisms of previously reported HfO_2_ thin films. Therefore, further investigation is needed to understand the wake-up effect and the fatigue mechanism.

In summary, the spatial distribution, concentration, and charge state of V_o_ in a HfO_2_ thin film strongly influence the polymorphism of HfO_2_. In the bulk region of HfO_2_, V_o_ help stabilize the polar orthorhombic phase. At the interface region, however, the lower concentration of V_o_ could produce a more stable polar orthorhombic phase. V_o_^2+^ with a positive charge state can contribute more to the stabilization of the polar orthorhombic phase than neutral V_o_. The stabilization of orthorhombic phase by the manipulation of V_o_ concentration is also strongly associated with the enhancement of the ferroelectric properties. The ferroelectric properties, such as P_r_ value and wake-up effect, can also be controlled by this approach. These results suggest that the design of V_o_ is key to improving the ferroelectricity of HfO_2_.

### Effect of V_o_ on reliability of HfO_2_

To apply HfO_2_ ferroelectrics to practical memory devices, the behavior of V_o_ under repetitive application of electric fields must be understood. Particularly, compared to perovskite ferroelectric materials (coercive field E_c_ = approximately 10–100 kV/cm), the high E_c_ of HfO_2_-based ferroelectrics (approximately 1,000–2,000 kV/cm) can sufficiently mobilize the V_o_ through the thin film [10, 63, 126–128]. Moreover, various reliability issues of ferroelectric HfO_2_ thin films, such as wake-up effect and fatigue, are closely related to the formation and migration of V_o_. Therefore, the behavior of V_o_ under the external electric field of the ferroelectric HfO_2_ thin film should be meticulously considered for the application of the HfO_2_ to the ferroelectric memory application.

The wake-up effect is a well-known reliability challenge associated with HfO_2_-based ferroelectrics. Per research, the redistribution of V_o_, which are initially concentrated at the interface of the HfO_2_ thin film, under electric field cycling and the subsequent phase transition from non-ferroelectric tetragonal phase to ferroelectric orthorhombic phase are plausible origins of the wake-up effect [63, 95, 100, 123–125]. Additional electric field cycling after the wake-up step causes a decrease in P_r_, which is known as fatigue [95, 96, 129]. One of the origins causes of fatigue could be the charge trap and ferroelectric domain-wall pinning due to the accumulation of V_o_ [96]. The wake-up effect and fatigue evidently influence the ferroelectricity and reliability of HfO_2_. In this section, therefore, the behavior of V_o_ in HfO_2_ thin films by external electric fields cycle and the evolution of ferroelectricity will be discussed.

**Fig. 6:**
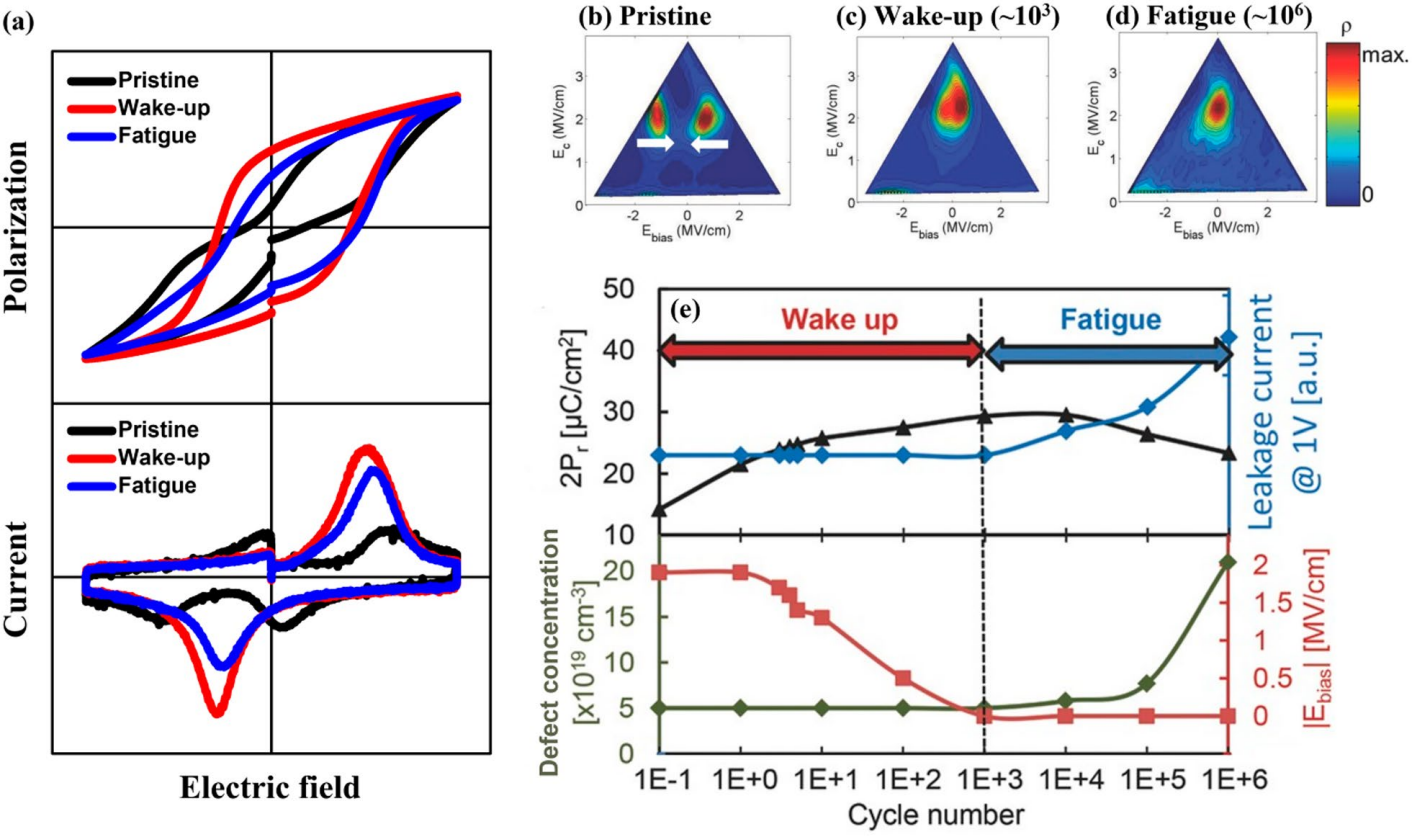
**a** Polarization–electric field and current–electric field curves of a HfO_2_ ferroelectric thin film for pristine, wake-up, and fatigue states. Evolution of the Preisach/switching density (ρ) of the HfO_2_ thin film after **b** ~ 1, **c** ~ 10^3^, and **d** ~ 10.^6^ cycles corresponding to the pristine, wake-up, and fatigue states, respectively. **e** Evolution of the 2P_r_, leakage current, defect concentration, and E_bias_ through repeated electric-field cycling. **b-e** reproduced with permission from [100]

Figure 6a illustrates the typical polarization–electric field and current–electric field curves of a HfO_2_-based ferroelectric thin film after wake-up and fatigue steps. In the pristine state, two distinct current peaks are observed, which are the origins of the pinched hysteresis loop. After electric-field cycling, however, the peaks merge and the hysteresis loop gradually opens and evolves into a single hysteresis loop. In the fatigue step, a decrease in the current peak intensity and P_r_ value were observed.

The wake-up effect, fatigue, and electrical breakdown are strongly correlated to the formation and migration of V_o_, so it is necessary to understand the behavior of V_o_ under electrical cycling. Pešić et al. discussed the migration of V_o_ under electric-field cycling using the first-order reversal curve (FORC) measurement technique [100]. The FORC measurement is an effective technique that can electrically analyze the redistribution of V_o_ by measuring changes in the electric field inside a material [130]. Figures 6b–d show the FORC measurement results of a TiN/Gd:HfO_2_/TiN capacitor after several electrical cycling. The results after 0, ~ 10^4^, and ~ 10^6^ cycles correspond to pristine, wake-up, and fatigue, respectively. In the pristine state, two separate Preisach/switching current density (ρ) peaks appear. These peaks are the origins of the two current peaks observed in the HfO_2_ thin film in the pristine state, as depicted by the current–electric field curve in Fig. 6a. The mechanism of this peak separation is the internal bias from the V_o_ concentration on a specific region (generally the interface region) [63, 100, 123]. After ~ 10^3^ electric-field cycles, which corresponds to the wake-up cycle, the two peaks merge into one. In addition, as shown in Fig. 6a, the pinched hysteresis loop opens and the two current–electric field curve peaks integrate. This means that V_o_, which had spatially inhomogeneity, was redistributed by repetitive application of electric field to fix the asymmetry of the internal bias. Additional electric-field cycling causes fatigue and a decrease in the intensity of the current–density peak. Considering the low P_r_ value of the fatigued state and the decline of peak intensity of the current–electric field curve in Fig. 6a, fatigue evidently degrades the ferroelectric properties of the HfO_2_ thin film. These results suggest that the migration of V_o_ under repeated external electric field cycling has a dominant effect on the ferroelectricity and reliability, such as P_r_ value, wake-up effect and fatigue, of HfO_2_ ferroelectric thin film.

Figure 6e highlights the variation of the electrical properties of HfO_2_ with the number of electric field cycles. The wake-up and fatigue steps correspond to the stage before and after ~ 10^3^ cycles, respectively. In the wake-up effect step, a gradual increase in P_r_ and a decrease in the internal bias (E_bias_) were observed. Conversely, after the fatigue step, the degradation of device characteristics deteriorates, such as a decrease in P_r_ value and an increase in leakage current density. These trends can be explained through the change and redistribution of defect (V_o_) concentration. In the wake-up step, the concentration of V_o_ is maintained at an approximately constant value. Meanwhile, the asymmetry of E_bias_ is gradually alleviated with the number of electric-field cycles. This is because no new V_o_ were produced during the wake-up step, and the V_o_ located at the interface region were redistributed into the bulk region of the HfO_2_ thin film. On the other hand, in the fatigue step, the concentration of V_o_ rapidly increases, and the internal bias did not change. This indicates the formation of new V_o_ in all regions of the thin film. An appropriate V_o_ concentration in the HfO_2_ thin film can stabilize the ferroelectric orthorhombic phases, whereas excessive V_o_ can rather degrade the stability of orthorhombic phases [99, 131]. Moreover, an increase in the leakage current density is related to the concentration of charge defects, such as V_o_ in ferroelectric thin films [131, 132]. In summary, the main mechanisms of the wake-up effect and fatigue in ferroelectric HfO_2_ films are the redistribution and additional formation of V_o_ under repetitive electric field, respectively. Electric-field cycling alleviates the inhomogeneous distribution of V_o_ and triggers the wake-up effect, which decreases the internal bias and increases the P_r_ value. Excessive electric-field cycles might cause fatigue with the additional generation of V_o_, which causes an increase in the leakage current and a decrease in the P_r_ value. As V_o_ strongly influences the ferroelectricity and electrical properties of HfO_2_, which are associated with changes in the concentration and migration of V_o_. Therefore, to stably apply the HfO_2_ thin film to a ferroelectric memory device, it is necessary to carefully control the concentration and migration of the V_o_.

The concentration, location, and migration of V_o_ have a strong effect on various properties such as ferroelectricity, polymorphism, and reliability of the HfO_2_ thin film. We will review suitable approaches for improving improve ferroelectricity by understanding the influence of V_o_.

### Strategies to control V_o_ of HfO_2_ and enhance ferroelectricity

As discussed in the previous sections, V_o_ in the appropriate concentration can stabilize and enhance the ferroelectric properties of HfO_2_, whereas excessive concentrations or asymmetric distribution of V_o_ can deteriorate the ferroelectricity and/or reliability. Therefore, the optimized fabrication and/or engineering method to induce the appropriate V_o_ concentration in HfO_2_ should be prudently considered. To apply ferroelectric HfO_2_-based thin film to the ferroelectric memory devices, strategies to control the V_o_ concentration in HfO_2_-based thin films have been proposed by several studies [133–138]. In this section, we review several previous studies that have reported improvement of ferroelectricity and reliability by controlling the concentration of V_o_ via various engineering idea.

**Fig. 7:**
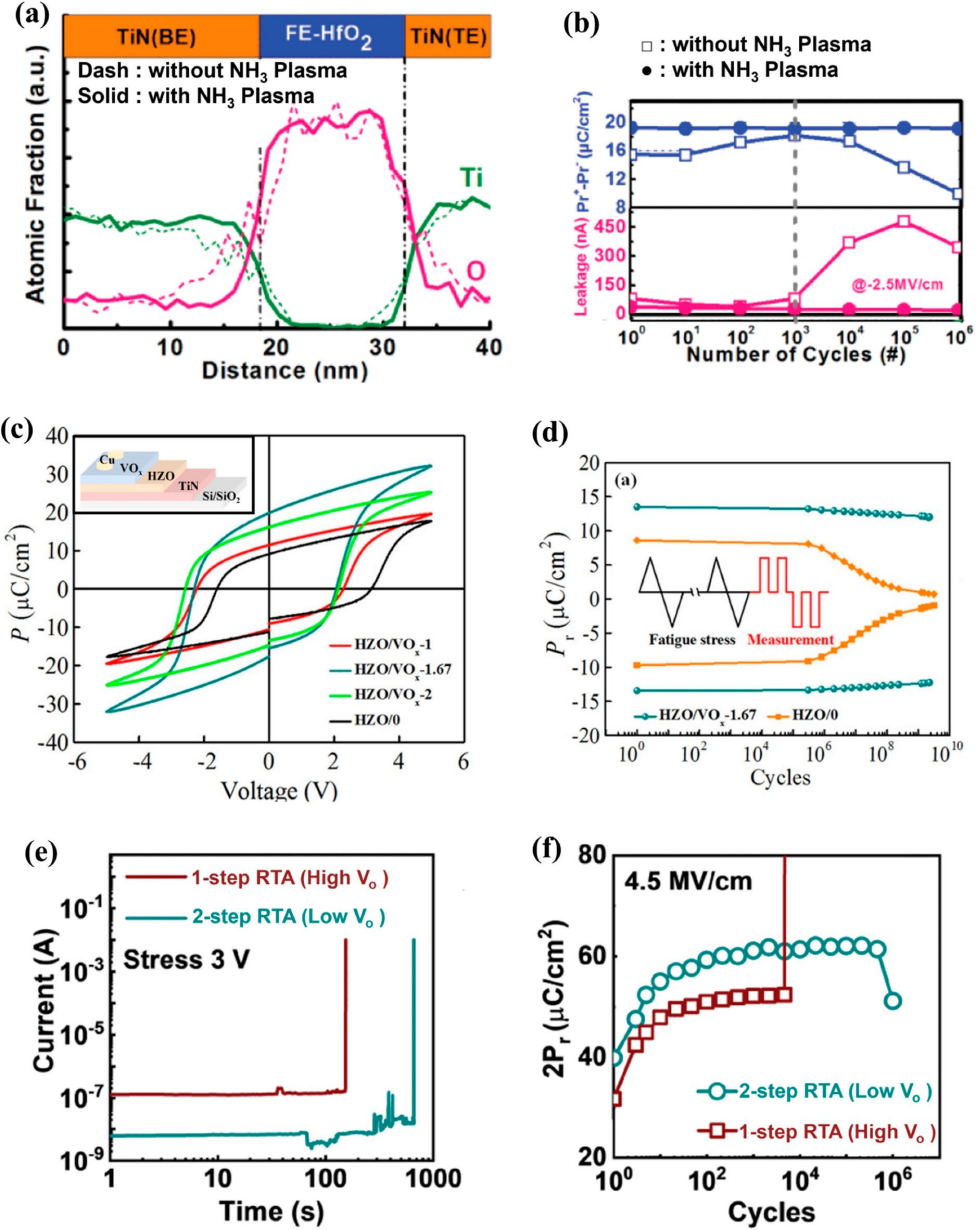
**a** EDS depth profile, **b** Evolution of P_r_ and leakage current as functions of number of cycles for TiN/Hf_0.5_Zr_0.5_O_2_/TiN capacitors with and without NH_3_ plasma treatment. **c** Polarization–voltage curves. **d** Endurance test of Cu/VO_x_/Hf_0.5_Zr_0.5_O_2_/TiN capacitor with various sol–gel solutions concentration. **e** Transient currents during constant voltage stress. **f** Endurance test of TiN/Hf_0.5_Zr_0.5_O_2_/TiN capacitor annealed with 1- 2-step RTA processes. **a**, **b** reproduced with permission from [137]. **c**, **d** reproduced with permission from [138]. **e**, **f** reproduced with permission from [145]

Chen et al. controlled the V_o_ concentration at the HZO thin film–TiN electrode interface by NH_3_ plasma treatment [137]. Figure 7a illustrates an EDS map of a TiN/HZO/TiN capacitor with and without NH_3_ plasma treatment. It is notable that NH_3_ plasma treatment increases the oxygen atom fraction on the interface region of HZO, while reducing the oxygen atom fraction of the interface region of TiN electrode side. This indicates that NH_3_ plasma treatment can decrease the V_o_ concentration on the HZO side of the interface. Figure 7b shows the evolution of various electrical properties with the number of electric-field cycles of ~ 10^6^ cycles for the HZO thin film with and without NH_3_ plasma treatment. The HZO thin film without NH_3_ plasma treatment exhibits the typical wake-up effect and fatigue. Over approximately 10^3^ cycles, the P_r_ value gradually increased, while the leakage current did not change significantly. This may be because of the redistribution of V_o_ without the formation of new V_o_, indicating the wake-up effect. After 10^3^ cycles, there are clear signs of fatigue, an increase in the leakage current and a decrease in the P_r_ value. On the other hand, the HZO thin film with NH_3_ plasma treatment exhibits low leakage current density as well as mitigated wake-up effect and fatigue. Consequently, treatment of HZO with NH_3_ plasma is a robust engineering approach for producing stable memory devices without drastic changes by electric-field cycles. This result is meaningful in that it demonstrates the improvement of reliability of HfO_2_ ferroelectric thin films via manipulation of V_o_ concentration.

Zhang et al. reported improvement in the ferroelectricity and endurance of the HZO thin film by inserting a vanadium oxide (VO_x_) capping between the TE and the HZO thin film [138]. Figure 7c illustrates the polarization–voltage curve of a Cu/VO_x_/HZO/TiN capacitor with a VO_x_ capping layer fabricated with sol–gel solutions (concentrations of 1, 1.67 and 2 mg/mL), respectively. The inset depicts the stack of the capacitor. Here, the samples are named HZO/VO_x_-1, HZO/VO_x_-1.67, and HZO/VO_x_-2, depending on the concentration of the sol–gel solution (1, 1.67, and 2 mg/mL, respectively) for VO_x_. The sample without a VO_x_ layer was named HZO/0. The highest 2P_r_ value of ~ 36.9 μC/cm^2^ was observed in the HZO/VO_x_-1.67. Figure 7d shows the endurance test results for HZO/0 and HZO/VO_x_-1.67. HZO/0 exhibits fatigue after ~ 10^6^ cycles and a deteriorated P_r_ value of ~ 0 μC/cm^2^ after ~ 10^9^ cycles. On the other hand, HZO/VO_x_-1.67 with the VO_x_ capping layer exhibited enhanced fatigue resistance, with a decrease in P_r_ of only ~ 10.9% even after ~ 10^9^ cycles. This result can be explained by the difference in the binding energy with various metal and oxygen. The V–O binding energy (625.0 ± 19.0 kJ/mol) energy is lower than those of Hf–O (810.0 ± 13.0 kJ/mol) and Zr–O (766.1 ± 10.6 kJ/mol). Hence, the VO_x_ capping layer could supply the oxygen atom to the HZO thin film and control the oxygen concentration [139]. Other studies have reported a similar phenomenon in HZO thin films using oxide electrodes, such as oxides of Ru and Mo [133]. MoO_3_ and RuO_2_ have lower formation enthalpy than HfO_2_ and ZrO_2_ from room temperature to RTA temperature (~ 800 ℃) [140–144]. By supplying oxygen atoms to the HZO thin film during thermal processes such as ALD and RTA, these oxide electrodes serve as oxygen supplier and thus control the V_o_ concentration. Therefore, by reducing the V_o_ concentration in the interface area of HfO_2_, the engineering technique of supplying oxygen atoms to HfO_2_ thin film can contribute to the enhancement of endurance properties and orthogonal stabilization.

Su et al. proposed a strategy to control the V_o_ concentration of the TiN/HZO/TiN capacitor by splitting the crystallization process into two sequential steps [145]. The 1-step RTA sample, which refers to the general RTA process for a HfO_2_ thin film, was conducted in an oxygen-free atmosphere at 600 ℃. Meanwhile, the 2-step RTA sample for controlling the V_o_ concentration was first crystallized at 600 ℃ and subjected to additional low-temperature heat treatment at 400 ℃ for approximately 30 min in O_2_ atmosphere. Figures 7e, f show the electrical stress resistance and endurance measurement results for the 1-step and 2-step RTA samples. For a constant voltage stress of 3 V, the electrical breakdown of the 2-step RTA sample occurred after that of the 1-step RTA sample.

Moreover, the 2-step RTA sample shows an ~ 10^2^ -times improved endurance cycle compared to the 1-step RTA sample. It was also attractive that the 2-step RTA sample exhibits a lower leakage current and a higher 2P_r_ value than the 1-step RTA sample for the same voltage stress time and number of electric-field cycles. An XPS analysis by Su et al. revealed that 1-step and 2-step RTA samples had V_o_ concentrations of 2.8% and 0.2%, respectively. This observation can be explained by the endurance of the relatively low V_o_ concentration by the 2-step RTA sample compared to that by the 1-step RTA under a longer electrical stress time. Furthermore, the increased P_r_ value and switching endurance indicate that the 2-step RTA process evidently contributed to the enhancement of ferroelectric and reliability properties of HfO_2_. This suggests that the 2-step RTA can improve the reliability of the HfO_2_ device without degrading its ferroelectricity and leakage current characteristics.

In summary, various engineering techniques can be implemented to regulate the V_o_ concentration in an HfO_2_ thin film for enhancing of P_r_ value, mitigating the wake-up effect and/or fatigue, and improving endurance. Strategies such as plasma treatment, artificial interface layer insertion, and RTA process improvement can effectively mitigate the nonideal effects of excess V_o_. If the endurance of the HfO_2_ thin films can be further improved without critically deteriorating P_r_ values, the HfO_2_ thin films could be a promising candidate for future ferroelectric memory applications.

## Effect of V_o_ in HfO_2_ base RS devices

The advantages of HfO_2_, which include its CMOS compatibility, low process temperature, high-k dielectric property, and process scalability, were extensively discussed and demonstrated in the literature [146]. These attributes establish HfO_2_ as a highly suitable material for a switching matrix in nonvolatile memory and neuromorphic computing applications, offering reliable and high-performance memory functionalities. The intrinsic factors of HfO_2_ can be seamlessly controlled, including the crystallinity, GB, stoichiometry, and oxide layer structure, through deposition or post-treatment processes because HfO_2_ is an established material that acts as a switching layer in V_o_-mediated RS devices among CMOS-compatible substances. In addition, extrinsic alterations such as doping, insertion of an interfacial layer, and engineering of oxide–electrode interface can manipulate the V_o_ dynamics for improving device performance.

However, the HfO_2_-based RS devices still need to be improved in terms of their performances such as operation energy, uniformity, gradual conduction modulation, and switching linearity for large-scale integration; the deliberate modulation of V_o_ dynamics and CF formation are crucial for optimizing HfO_x_-based RS devices [147–155]. Therefore, numerous studies have been conducted for improving the uniformity and multilevel RS characteristics of HfO_x_-based RS devices via different engineering approaches. In Sect. 4.1, the valance change mechanism (VCM), which is a filament-evolution mechanism based on V_o_ dynamics, is described. In Sect. 4.2, the effect of V_o_ on VCM-based RS device performance is discussed. Based on the results, Sect. 4.3 introduces the various engineering strategies that have been utilized for controlling the V_o_ concentration in HfO_2_ and improving the RS characteristics.

### Valance change mechanism (VCM)

The switching behavior of RS devices is related to the various phenomena of CF evolution. The CF evolution occurs via electrochemical metallization (ECM) or VCM, which depends on the materials that compose the CF [156–160]. In HfO_2_-based RS devices that lack an active electrode for delivering highly mobile cations, the V_o_-mediated VCM is of vital importance for both device operation and CF formation. V_o_ are defects in the crystal lattice with missing oxygen atoms, which result in a change in the valence state of the nearby hafnium atoms. Thus, the presence, electromigration, and aggregation of V_o_ enable the formation of conductive paths within the insulating HfO_2_ material, which in turn produces the switching behavior observed in RS devices.

**Fig. 8:**
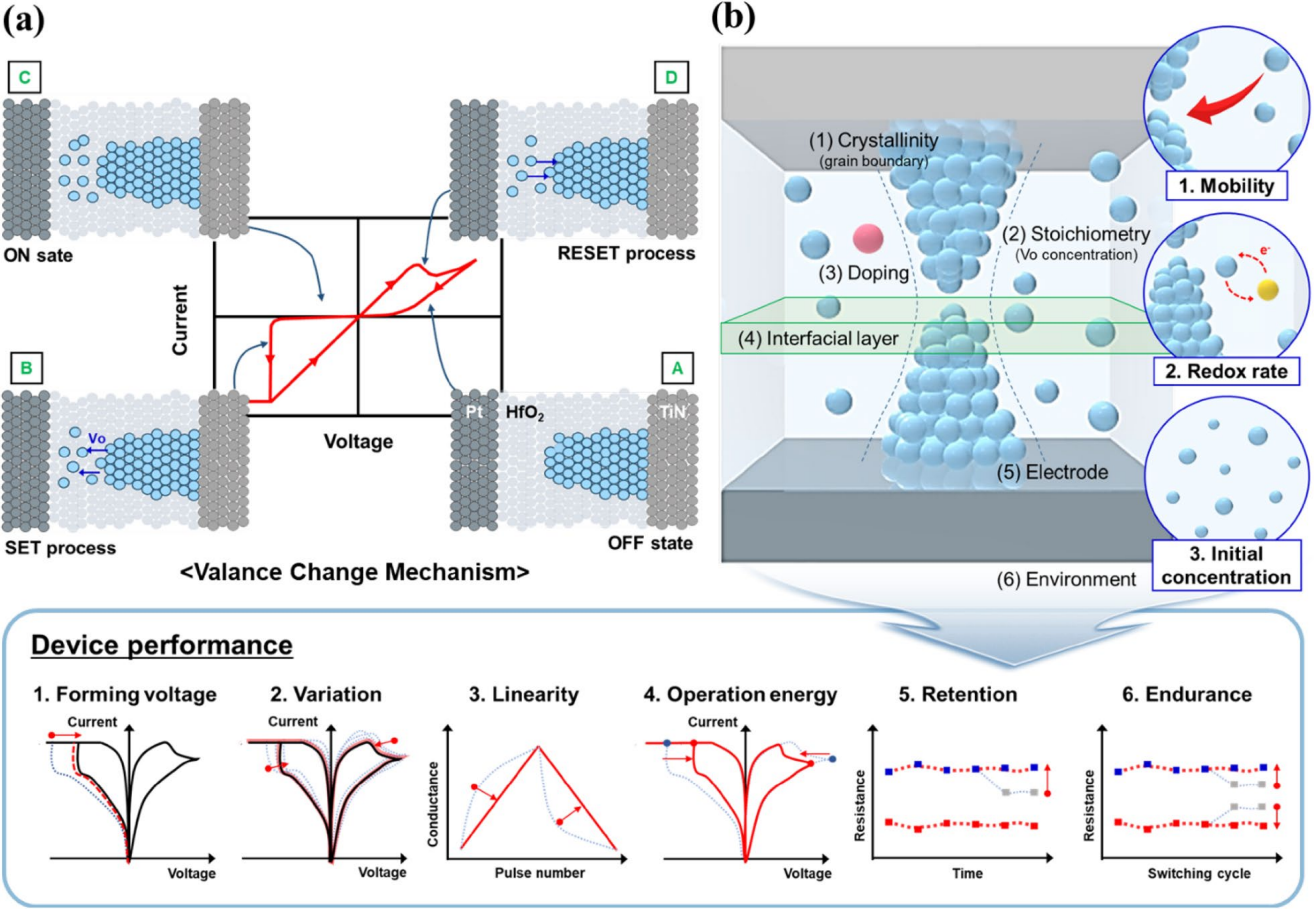
**a** Schematic of the working principle of a VCM device. **b** Various strategies to control the effect of V_o_ properties on the metrics of the RS device performance

The switching mechanism of VCM-driven RS devices is associated with the formation, migration, aggregation, and rearrangement of V_o_ [21, 79]. The VCM is commonly observed in metal oxides that exhibit a remarkable mobility of ions within the crystal lattice, which includes the migration of oxygen ions. The migration of these ions alters the local stoichiometry of the switching layer, thereby inducing a valence change in the cation sublattice and a change in electronic conductivity. V_o_ can be deliberately introduced for operating RS devices by applying a specific set of electrical pulses. These pulses are referred to as “forming” pulses and used to initialize the device by generating a CF. The forming process involves the migration of V_o_, which cluster to form a conductive pathway [161]. Once the CF has been formed, the movement of V_o_ becomes crucial for the subsequent switching operation. Under the application of an external electric field, V_o_ can migrate within the switching layer, which is facilitated by the presence of defects, GBs, or other structural irregularities in the material. Owing to the electrostatic forces, V_o_ tend to migrate toward the negatively biased electrode (cathode), which accumulates at the interface between the HfO_2_ layer and the electrode to form a CF. This establishes a low-resistance pathway by bridging the electrodes, i.e., switching ON the cell (LRS). The cell can be switched OFF (HRS) by repelling V_o_ from the electrode by applying a positive voltage. The evolution of CF with V_o_ migration dynamics at each operational stage of the switching mechanism is illustrated in Fig. 8a.

### Effect of V_o_ on RS device performance

The presence of V_o_ in an RS device has a significant effect on its multiple performance aspects. It plays a crucial role in forming and dissolving CFs, which directly affects the switching behavior between HRS and LRS. The migration and accumulation of V_o_ affect the stability and endurance of the device, as well as the switching speed and voltage. In this section, the device performance is explicitly divided into various metrics and the influence of V_o_ on them is analyzed [162, 163].

#### Forming voltage

The forming voltage is the initial voltage required to create a conductive pathway for operating an RS device. For VCM-driven RS devices, the resistance value in the HRS is significantly affected by the V_o_ generated during the forming process [164, 165]. A high forming voltage leads to the generation of excess V_o_, thereby forming a strong CF and exhibiting higher current levels in the HRS. Furthermore, the forming process induces large device-to-device variation, making the switching behavior unreliable. As V_o_ plays an important role in facilitating the formation of CFs by providing mobile charge carriers, the forming voltage depends on the concentration of the intrinsic V_o_ of the switching layer and other extrinsic factors, which include electrode material, interfacial layer, and dopants. That is, CF-free RS devices can be designed with appropriate switching materials and device structures to improve V_o_ controllability.

#### Variation

The nonuniform distribution of V_o_ in the switching matrix, which results in different filament paths and resistive states, causes performance variation [166, 167]. Fluctuations in the V_o_ concentration affects RS parameters such as the resistance level, switching voltage, and endurance characteristics; this results in device-to-device and cell-to-cell variations. Studies have explored various techniques, which include optimizing the deposition and distribution of V_o_ within the resistive layer, controlling the fabrication process parameters, and developing materials with smoother V_o_ profiles for addressing these nonuniformities and improving the device performance. The device-to-device, cell-to-cell, and cycle-to-cycle variations in V_o_-mediated RS devices can be reduced by achieving more precise control over the V_o_ distribution. Knowledge and regulation of the distribution of V_o_ can help mitigate these nonuniformities and improve the overall performance and reliability of RS devices.

#### Switching behavior

Switching behavior can be classified into abrupt and gradual behaviors based on its characteristics. While abrupt switching is characterized by a fast switching speed, it is limited in terms of endurance and device-to-device variation caused by the nonuniform formation and rupture of CFs [168, 169]. The transition from the abrupt to gradual RS behavior is influenced by the quantity of V_o_ in the switching layer [170, 171]. The transition between the two resistance states is sudden and discontinuous when the layer consists of fewer V_o_, which results in abrupt switching behavior. The limited number of V_o_ restricts the movement of ions and the formation of CFs, leading to a distinct and rapid change in resistance. Conversely, when the layer contains sufficient V_o_, the RS behavior becomes gradual because a higher concentration of V_o_ enables a more continuous transition between HRS and LRS. This gradual switching behavior is due to the enhanced ion migration and CF formation, allowing for an analog change in resistance. That is, the availability of V_o_ directly influences the switching behavior in RS devices.

#### Switching speed and switching voltage

The migration of V_o_ and formation/dissolution of CFs determine the switching speed between HRS and LRS. The presence of sufficient Vo facilitates the formation and rupture of CFs, enabling faster transitions between different resistive states and faster switching speeds [172]. Moreover, the concentration and distribution of V_o_ influence the switching voltage required to trigger the RS operation. The higher concentrations of V_o_ tend to lower the switching voltage of the device because of the increased availability of charged species, such as oxygen ions, which can migrate more easily in the presence of V_o_. Thus, a lower voltage is required to initiate the movement of ions and the formation of CFs, enabling RS. Therefore, controlled concentrations of V_o_ can enhance the switching speed by promoting ion movement and filament formation. In addition, higher V_o_ concentrations can reduce the switching voltage required for RS.

#### Conductance modulation

The development of RS devices that exhibit linear conductance modulation is pivotal for their applications in multilevel memory or neuromorphic computing systems. The enlargement or disruption of V_o_ CFs in an RS device is governed by two mechanisms with different growth and dissolution rates: a fast redox process and a slow V_o_ diffusion process [173, 174]. RS devices exhibit nonlinear conduction modulation under the influence of these two processes with different reaction rates. The abrupt variation of conductance results from the inhomogeneous growth/dissolution of V_o_ filaments, which is in turn caused by the simultaneous involvement of the two mechanisms with different growth/dissolution rates. In this context, the growth/dissolution of CF should be controlled by either mechanism to improve the linearity of conductance modulation. The diffusion reaction becomes the predominant growth mechanism of V_o_ CFs when only a few V_o_ are present [175]. In contrast, the redox reaction takes precedence when numerous V_o_ exist [173]. Hence, the number of V_o_ in the RS device must be controlled so that only one mechanism is involved in the growth of CF to ensure linear conductance modulation.

#### Endurance and retention

Endurance is a crucial factor in ensuring reliable and long-lasting device performance. During the SET/RESET switching cycle in RS devices, the irreversible defect generated by excess V_o_ accumulation deteriorates the oxide layer, which leads to device failure. The generation of irreversible defects in the switching layer must be curtailed because the accumulation of additional V_o_ leads to failure with the generation of new CFs [169, 176]. Moreover, the retention properties of RS devices, which reflect their ability to retain stored information, are influenced by various factors, including device structure, materials, and current. Particularly, the activation energy, which is determined by the energy of the highest transition state during the V_o_ diffusion process, is a critical factor for the retention behavior [177].

In summary, exploiting the technical solution to control the V_o_ dynamics is vital for improving device performance. Therefore, the engineering approach for improving the performance of the HfO_2_-based RS device, which involves intrinsic factors (crystallinity, stoichiometry) and extrinsic factors (doping, interfacial layer, electrode, and environment) (Fig. 8b) is discussed in the following section.

### Strategies to control the V_o_ of HfO_2_ and enhance RS characteristics

#### Crystallinity (GB)

**Fig. 9:**
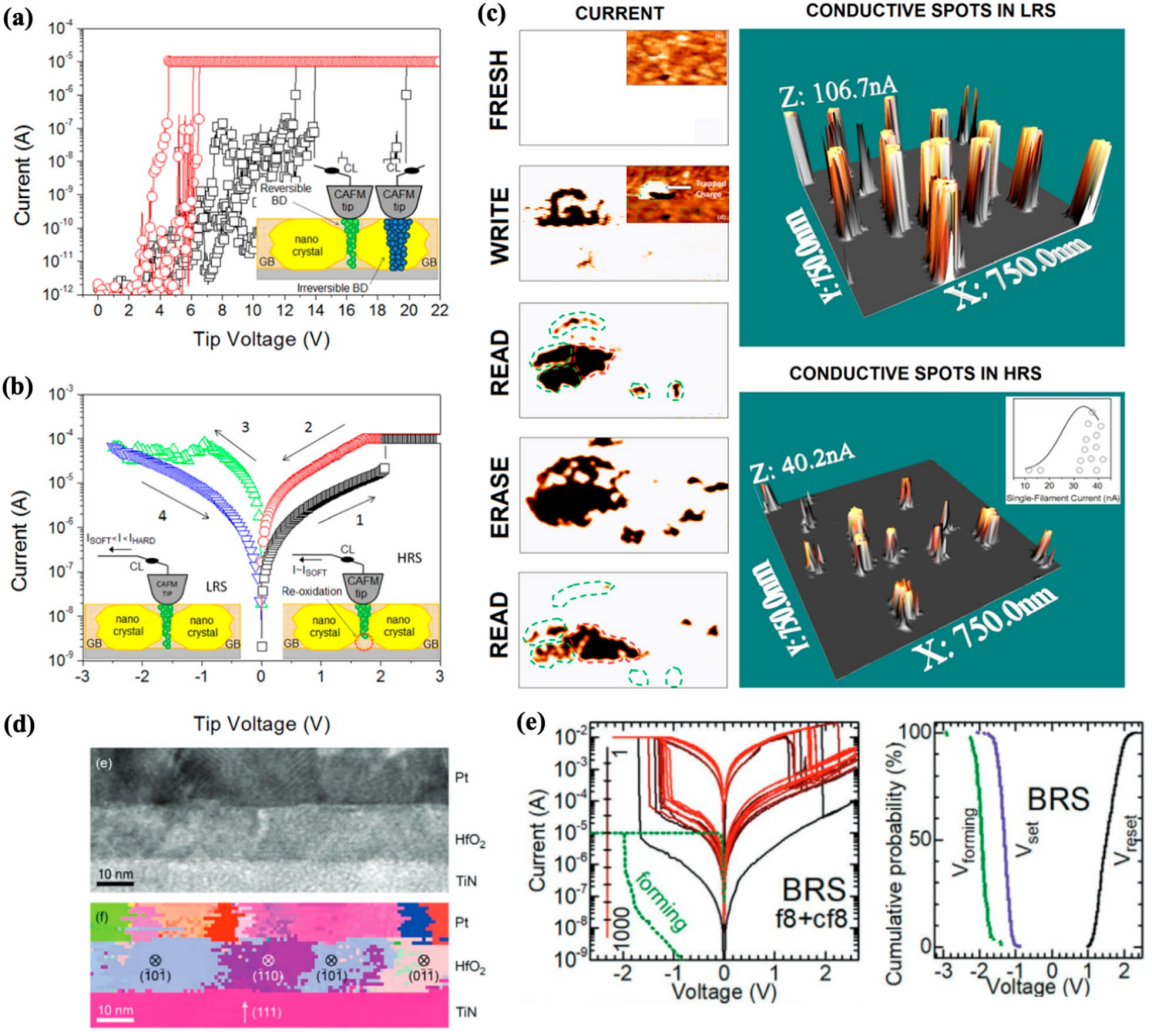
Low forming voltage exhibited on low-energy GBs in HfO_2_-based RS devices. **a** Forming process in annealed polycrystalline HfO_2_ at specific locations. **b** Typical bipolar RS behavior observed at the low voltage forming site (GB location). **c** Current map measurement in a write–read–erase–read cycle, which allows the locations to recover their insulating properties. **a**, **b**, **c** reproduced with permission from [184]. **d**, **e** reproduced with permission from [187]

The crystallinity of HfO_x_ thin films, which includes the size and shape of the grains and the presence of GBs, is one of the most significant factors influencing the V_o_ motion dynamics for RS behavior. In the early stage of the V_o_-mediated RS research on HfO_2_, several studies were conducted to identify the active switching region where the V_o_ is generated, migrated, and agglomerated, and thus formed CFs. Therefore, several theoretical and experimental studies investigated the relationship between the crystallinity of HfO_2_ and the V_o_ motion dynamics and its RS behavior. Most theoretical studies were based on DFT calculations [83, 85, 178, 179], suggesting that GBs in the crystalline HfO_2_ layer were the preferred locations for generating and migrating V_o_ caused by the lower diffusion barrier for oxygen ions (vacancies) diffusion [149]. Based on the Ab initio model, Bersuker et al. demonstrated the evolution of a current pathway at the GBs in monoclinic HfO_2_ during the forming process [84]. In their study, pre-existing V_o_ were energetically favored to segregate near the GBs, eventually forming the preferential leakage current pathway [180–182]. This conjecture from theoretical calculations was verified by c-AFM experiments. By comparing the electrical and topographical data of HfO_2_ stacks in amorphous and polycrystalline HfO_2_ phase thin films, Lanza et al. analyzed the effects of crystallinity on the RS behavior [178, 183, 184]. In the case of amorphous HfO_2_, leaky sites were arbitrarily distributed throughout the measured area. In contrast, the current increased at the area with the GBs of crystalline HfO_2_, indicating a correlation between topographic features and output current. When a voltage is applied to the c-AFM probe tip at a random spot, it induces two types of electroforming processes. In most regions, the process occurs with a high voltage (> 12 V), while in specific regions, such as the GBs, it occurs at significantly lower formation voltages (4 < V < 6.5). (Fig. 9(a)). After the high-voltage forming process, no RS behavior was detected in the subsequent I–V curves for those most regions. However, in areas where the forming voltage is low, a typical bipolar RS behavior is observed (Fig. 9b). When the probe tip was placed on the grain, it required a high voltage to continue the forming process, resulting in a hard dielectric breakdown of the device. Conversely, when the probe was placed on the GB, it required a low forming voltage to generate the CFs without causing a dielectric breakdown of the device. In addition, the study captured the topographic and output current profiles after iteratively performing the write–read–erase–read operations. The profiles confirm that the conductive pathways at the GBs can be erased through the RESET operation (Fig. 9c).

The degree of crystallinity and the GB of the HfO_2_ thin film share strong correlations with the RS properties. Several studies have explored methods to enhance the RS functionality by controlling the degree of crystallinity and the GBs in HfO_2_ thin films [83, 185, 186]. Petzold et al. demonstrated the development of forming-free RS devices by engineering the GBs of HfO_2_ [187]. The study focused on the influence of V_o_ with respect to the GB and fabricated a device based on an epitaxial stack combination of TiN (111) and monoclinic HfO_2_ (11 $\overline{1 }$) in TiN/HfO_2_/Pt stacks. They grew TiN and HfO_2_ layers on a (0001)-oriented Al_2_O_3_ substrate using molecular beam epitaxy (MBE). This resulted in a defined subset of GBs with high symmetry (Fig. 9d). The electrical characterization of the devices reveal that they exhibit reliable and forming-free RS behavior (Fig. 9e). According to the study, the uniform distribution of forming and switching voltage might have emerged from the formation of CFs on a predefined path formed along the low-energy GBs, which would require a low forming voltage, thereby forming uniform filaments. According to these studies, GBs can be used to precisely control the V_o_ motion dynamics, resulting in improved RS performance. However, the randomized GBs in each cell can impact the variability of the forming voltage, which can hinder the device-to-device uniformity. Hence, the formation of a well-defined GB connecting the TE and BE is considered important for precisely controlling the V_o_ in RS devices [188, 189].

#### Stoichiometry (Oxygen deficiency)

**Fig. 10:**
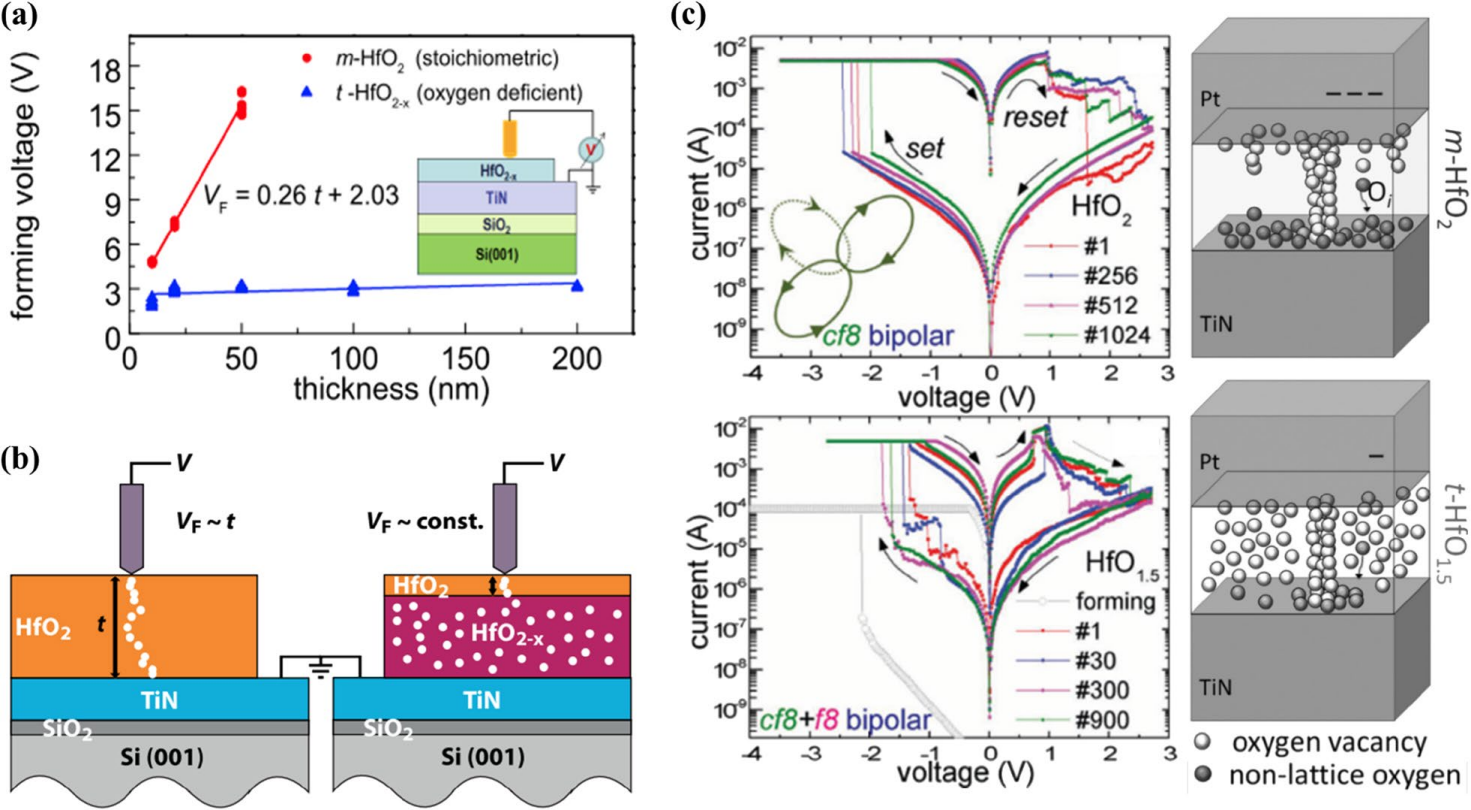
Reduced forming voltage in oxygen stoichiometry-engineered HfO_2_-based RS devices. **a** Forming process in annealed polycrystalline HfO_2_ according to the specific location. Low variation of forming voltage with thickness for oxygen deficient HfO_2–x_ film. **b** Model for the thickness dependence of forming voltage in stoichiometric (left) and oxygen deficient HfO_2–x_ (right). **c** Switching characteristics tuned in oxygen deficient t-HfO_2–x_. Schematic model of filament formation in oxygen engineered HfO_x_-based RS devices. **a**, **b** reproduced with permission from [228]. **c** reproduced with permission from [12]

The stoichiometry of the HfO_x_ material layer can be adjusted by controlling the deposition condition during the fabrication process or during post-processing. This significantly influences the RS characteristics as it requires adjusting the concentration of V_o_ (oxygen deficiency) in the RS layer. Therefore, stoichiometry must be considered in addition to the V_o_ motion dynamics induced by the voltage application during RS operation. Several studies have indicated that a well-controlled oxygen stoichiometry determines the formation of V_o_ CFs [190, 191]. Employing oxygen engineering, Kaiser et al. identified the oxygen-dependent phase transitions from stoichiometric hafnia (m-HfO_2_) to hexagonal phase hcp-HfO_0.7_ [192]. Oxygen vacancies were introduced into the crystalline matrix with a decrease in oxidation conditions, thereby forming V_o_ defects. Therefore, through oxygen engineering, a V_o_-driven significant decrease in resistivity from the insulating m-HfO_2_ (~ 10^10^ Ωm) over cubic c-HfO_1.7_ (~ 10^–4^ Ωm) to hexagonal hcp-HfO_0.7_ ($\sim 7.3\times {10}^{-6}$ Ωm) is induced. McKenna et al. emphasized the importance of sub-stoichiometric HfO_x_ in ensuring the uniform nucleation and growth of CFs during the forming process [193]. Through a first-principles study, they identified that an optimal HfO_x_ stoichiometry with x in the range 1.50–1.75 was necessary for the efficient nucleation and growth of stable CFs during the forming process. The activation energy required for the nucleation of the CF is reduced within this optimal stoichiometric range, thereby facilitating the formation of clusters that serve as seeds for CF growth. Further, the DFT calculations demonstrated the stability of Hf-rich precipitates and their growth propensity by the outward diffusion of oxygen ions. This supplemented additional evidence that the range of 1.50–1.75 was conducive to the formation of CFs. In addition, the relationship of the forming voltage with the stoichiometry of HfO_2_ was investigated by comparing the cases of the stoichiometric (m-HfO_2_) and oxygen-deficient (t-HfO_2–x_) films [194]. Both films were epitaxially grown by reactive molecular beam epitaxy (RMBE) under different oxidation conditions. Figure 10a shows the forming voltage of oxygen-deficient devices, which are composed of sub-stoichiometric HfO_2-x_, is independent of film thicknesses up to 200 nm; however, the forming voltage of stoichiometric HfO_2_-based devices increases linearly with the thickness after overcoming an initial energy threshold to activate the ionic transport for CF formation. In the sub-stoichiometric HfO_2–x_, which is a layer with a thin oxidized top layer (Fig. 10b), the forming voltage was constant because only one filament needs to be formed in the oxidized top surface layer. The reason for the constant forming voltage is that the surface layer maintains a constant thickness because of using the same sample treatment method. These results show that a highly oxygen deficient layer has sufficient conductivity and does not contribute to the increase in forming voltage until the total oxide layer thickness is increased to 200 nm. Consequently, the oxygen-deficient layer acts as a V_o_ reservoir by employing well-defined oxides with controlled oxygen stoichiometry as the switching layers, which enables the implementation of forming-free switching behavior.

Sharath et al. used MBE to precisely engineer different phases of HfO_2_, including hcp-Hf(O), t-HfO_x_, and m-HfO_2_, with varying oxygen stoichiometries [12]. The RS devices using the t-HfO_x_ and m-HfO_2_ phases exhibited distinct forming and switching operations (Fig. 10c). In t-HfO_x_-based devices, intrinsic V_o_ caused a significant reduction in the forming voltage. Meanwhile, in m-HfO_2_, a higher number of Hf–O bonds were broken to generate V_o_, which resulted in a higher forming voltage (V_F_ ≈ − 7.0 V) and stronger filaments with more accumulation of oxygen ions near the TiN interface. Unformed and pristine t-HfO_1.5_ already contains a homogeneous distribution of numerous V_o_, and therefore, the filament is formed under a considerably weaker electric field (V_F_ ≈ − 2.2 V), where only a few Hf–O bonds needed to be broken because of the presence of pre-existing V_o_.

The stoichiometric control of HfO_2_ in RS devices has significant implications for improving their RS properties. The literature demonstrated that the precise engineering of the oxygen stoichiometry in HfO_x_-based metal–insulator–metal structures can help significantly reduce the forming voltage. Consequently, comprehending the material conditions and RS operation aids in establishing the correlation between the structural and functional properties of RS device materials. These approaches enable the development of reliable RS devices by leveraging the ability to engineer the oxygen stoichiometry in HfO_x_.

#### Doping

In addition to the intrinsic properties of the HfO_2_-based RS layer, such as crystallinity and stoichiometry, extrinsic factors can be engineered to change the properties of the layer. These factors facilitate the highly controllable V_o_ dynamics to improve the RS performance. The V_o_ motion dynamics must be modulated to precisely control the rupture and reformation process of V_o_-based CFs in the HfO_2_ RS device during operation. Several studies have proposed a few viable solutions, including element doping, metal/oxide interface modulation, and multilayer structures.

**Fig. 11:**
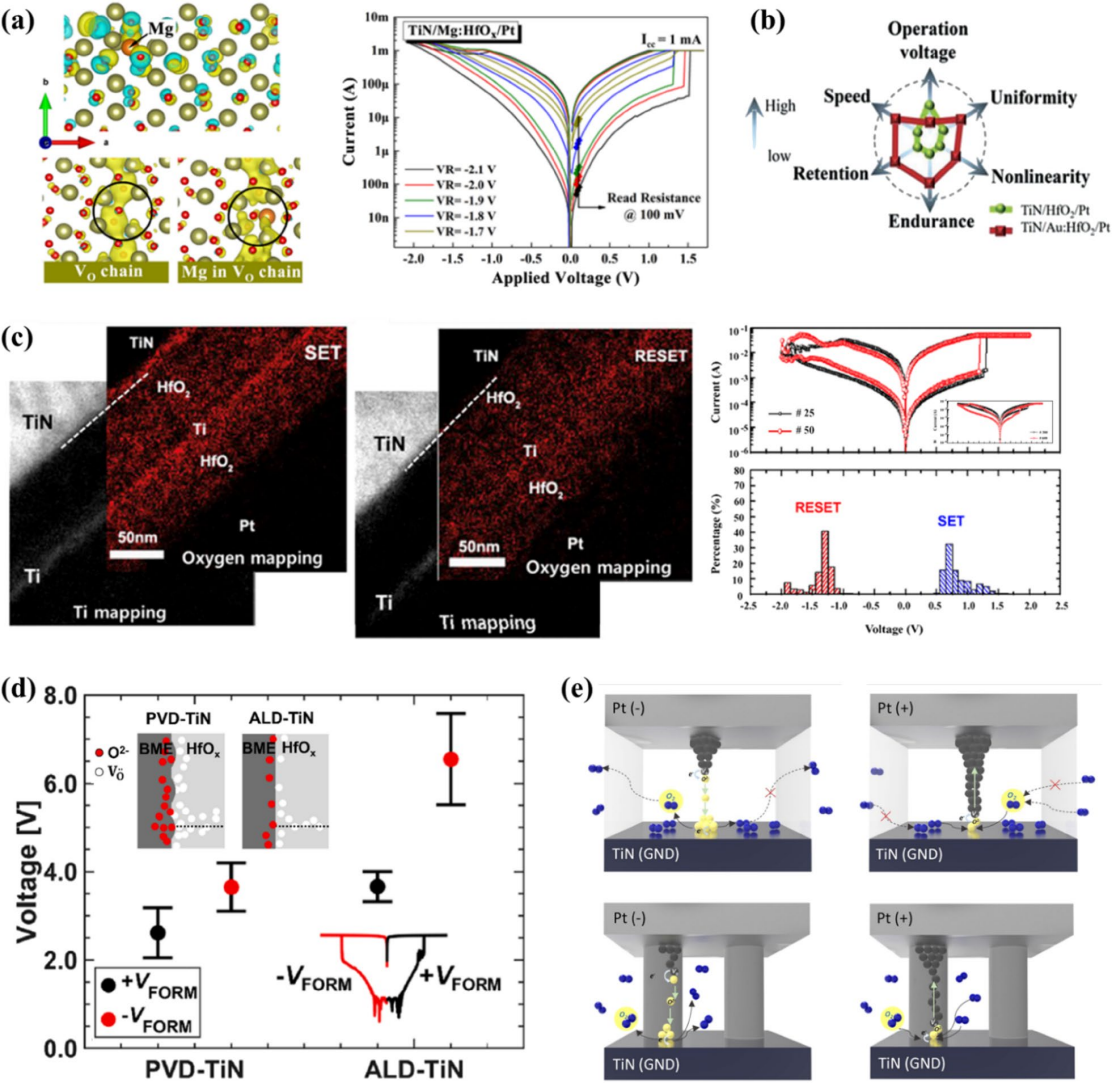
Doping and interfacial engineering of HfO_2_-based RS devices. **a** Mg doping to modify V_o_ migration kinetics in HfO_2_. The multilevel I–V cycles with varying reset voltages of TiN/Mg:HfO_x_/Pt devices. **b** Improvement in performance of HfO_x_-based RS device using doping. **c** Enhanced switching characteristics for TiN/HfO_2_/Ti/HfO_2_/Pt/Ti stack RS devices, which use Ti as the interlayer. **d** Forming voltage distribution for PVD-TiN (left) devices and ALD-TiN (right) devices. **e** Structural modulation of HfO_2_ switching matrix as nanorod structures for exploiting the environment as the V_o_ reservoir. **a** reproduced with permission from [195]. **b** reproduced with permission from [199]. **c** reproduced with permission from [213]. **d** reproduced with permission from [215]. **e** reproduced with permission from [217]

Zhao et al. utilized FT calculations to investigate the effect of metal dopants in these devices [57], and they analyzed the interaction between metal dopants and V_o_ based on formation energy. Consequently, they discovered that *p*-type metal dopants had a considerable effect on the formation of V_o_ CFs. Unstable metal–oxygen bonds were formed because of the lack of valence electrons in the *p*-type metal dopants, which in turn generated V_o_ around the dopants. In addition, metal dopants facilitate control over the mobility of V_o_ via the modulation of the activation barrier. Li et al. investigated the effect of Mg doping in HfO_x_ by comparing TiN/HfO_x_/Pt and TiN/Mg:HfO_x_/Pt memristors [195]. The comparison showed that the Mg dopant tended to regulate the activation barrier energy of the adjacent V_o_ and alter the defect levels in monoclinic HfO_2_ [196]. This implies that a mutual-ion effect might occur between Vo^2+^ and Mg^2+^ during the RS operation when HfO_x_ is doped with Mg. As shown in Fig. 11a, when Mg^2+^ migrates toward the V_o_ chain, it perturbs the consecutive channel of the original defect levels. The study found that regulation of the rate of V_o_ migration and the defect states of CFs enhance the controllability of the RS operation. Lee et al. investigated the doping effects of various aliovalent ions (Mg^2+^, Al^3+^, Nb^+5^) into HfO_2_ on RS characteristics [197]. Doping with aliovalent elements caused an increase in the nonlattice oxygen concentration and a reduction in the grain size in HfO_2_. The RS characteristics of the doped HfO_2_ are significantly influenced because the grain boundaries can act as favorable diffusion paths for atomic diffusion; therefore, the characteristics exhibit low forming voltage and improved uniformity in doped-HfO_2_ films. Moreover, Roy et al. demonstrated the beneficial effects of Al doping on the enhancement of the HfO_2_-based memory device performances [198]. They investigated the effect of Al doping on the formation of V_o_, demonstrating that the 16.5% Al doping concentration enhances the RS properties of the device. The loss of oxygen in the HfO_2_ layer is induced with an increase in Al doping concentration, which increases the formation of V_o_ and decreases the forming voltage. The comprehensive experimental analysis involving TEM and operando HAXPS indicated that Al-doping enhances the formation of V_o_ in HfO_2_ and improves RS performances, thereby demonstrating the achievement of synaptic simulation. The effect of doping on RS performance was demonstrated with a Au-nanoparticle-doped HfO_2_-based device, in which the metal nanoparticles/crystals were embedded in a HfO_2_ layer [199]. Wu et al. explained that the particles induce defects (V_o_) inside the oxide layer when metal particles were embedded in HfO_x_. These particles can enhance the local electric field, which is also a key factor in reducing the forming voltage [200, 201]. The improved overall device performance metrics are summarized in Fig. 11b.

Hence, studies demonstrated the effect of doping on V_o_ motion dynamics using various factors, including the energy required to generate or dissipate V_o_ in the HfO_2_ lattice, V_o_ mobility for migration, and intensity of the localized electrical field in the HfO_2_ layer. As the specific impact of doping on V_o_ in HfO_2_-based RS devices depends on different factors such as the dopant species, concentration, distribution, and the overall device design, experimental investigations need to be conducted to determine the optimal doping conditions that enhance the RS characteristics.

#### Interfacial layer

The incorporation of interfacial layers, such as 2D materials or other buffer layers, into the metal/oxide interface is a breakthrough approach for optimizing the interface properties in RS devices [202–204]. 2D materials were widely employed as interfacial layers in RS devices to reduce power consumption and mitigate detrimental surface effects [202, 205]. For example, graphene has been frequently employed as an interfacial layer to block atomic diffusion between the metal and the oxide. Zhu et al. used first-principles calculations to investigate the mechanism of the integration of graphene into the Au/HfO_2_ interface for enhanced interface properties [206]. They discovered that graphene influenced V_o_ diffusion by preventing strong interactions between the Au electrode and the HfO_2_ layer. This interfacial graphene effectively prevented the interface from becoming metallic and improved the dielectric reliability of HfO_2_. However, the study did not experimentally demonstrate the improvement of RS characteristics according to the graphene interfacial layer. Mannequin et al. experimentally investigated the improvement of RS properties in TiN/Pt/HfO_2_/graphene/Au stacks [207]. They discovered that the graphene interfacial layer acted as an oxygen reservoir, stabilizing oxygen ions released during the SET operation, which consequently improved the ON state retention.

The integration of a metal interlayer between the HfO_2_ layer and an electrode can impact the V_o_ behavior in different ways [208]. First, the metal interlayer can act as a diffusion barrier, limiting the migration of V_o_ from the HfO_2_ layer toward the electrode. This confinement of V_o_ within the HfO_2_ layer can stabilize the CF formation and improve the switching uniformity and endurance of the device. Moreover, the metal interlayer can facilitate oxygen exchange between the HfO_2_ layer and the electrode. Depending on the materials involved, it can capture oxygen from the electrode side or provide oxygen to the HfO_2_ layer. This exchange process influences the V_o_ concentration within the HfO_2_ layer, which will influence the RS characteristics. In addition, the metal interlayer can react with the HfO_2_ layer or the electrode material at the interface. These reactions can alter the local V_o_ concentration, modify the electrical properties at the interface, and subsequently impact the RS behavior. For example, utilizing Ti as the oxygen-gettering layer to produce TiO_x_/HfO_x_ helped engineer highly reliable HfO_2_-based RS devices with exceptional performances [209–212]. Further, the required forming voltage and set voltage were significantly reduced. Lee et al. investigated the characteristics of RS in TiN/HfO_2_/Ti/HfO_2_/Pt/Ti stacks [213] and demonstrated that embedding reactive metallic layers, which act as an oxygen buffer layer, into the HfO_2_ films can effectively optimize the RS behavior. The employed TEM and EELS mapping and directly observed the accumulation of oxygen ion at the Ti/HfO_2_ interfaces and successfully improved the RS characteristics, such as a lower SET voltage and larger memory window (Fig. 11c). Furthermore, the significance of the position of the Ti layer was demonstrated [208]. By integrating the Ti adlayer on the top and bottom of the HfO_2_ layer in TiN/HfO_2_/TiN device stacks, Walczyk et al. investigated the impact of the Ti layer’s deposition position in RS operation. The XPS depth profile data in that study revealed that a Ti layer deposited on the top of HfO_2_ effectively attracts oxygen at the interface, and when deposited on the bottom of HfO_2_, the layer exhibits lower reactivity in attracting oxygen. This is because the Ti bottom adlayer was terminated by oxygen when the Hf(NMeEt)_4_ precursor was introduced and oxidized during atomic vapor deposition, thereby reducing the reactivity of the layer with the subsequently grown HfO_2_ film. However, as additional Ti layers are deposited on the top of the HfO_2_ layer, Ti atoms and clusters with unsaturated bonds are deposited, thereby increasing its affinity toward oxygen from HfO_2_. Consequently, the deposition of the Ti top adlayer led to the formation of a nonstoichiometric Ti/HfO_2–x_ interfacial structure, which played an important role in achieving reliable RS.

The interfacial layer in HfO_2_-based RS devices can affect the V_o_ concentration and motion within the HfO_2_ layer through oxygen diffusion barrier effects, exchange processes, and interfacial reactions. These modifications can improve the RS characteristics, such as enhanced switching uniformity, stability, and endurance. These investigations highlight the criticality of interface management in influencing device performance. Establishing well-defined approaches for manipulating the physical and chemical characteristics of the interfacial layer/HfO_2_ interface is integral to attaining consistent and reliable RS performances.

#### Electrode

The choice of electrode material is vital for enhancing the controllability of V_o_ dynamics. Several studies have investigated the effect of the electrode material on RS operations. Padovani et al. employed Ti metal electrodes as a reactive buffer layer on the anodic side to attract oxygen atoms from the HfO_2_ layer, which promoted the formation of a sub-stoichiometric HfO_2_ region and improved the forming process in the RS operation [214]. This approach oxygen atoms to diffuse from the HfO_2_ layers to the Ti metal electrode, which in turn resulted in substantial oxygen deficiency in HfO_x_ (x ≈ 1.4). The oxygen in the HfO_2_ layer shifted to the Ti layer and created an HfO_2–x_ layer at the interface because Ti exhibits high oxygen-gettering activity. This Ti-induced sub-stoichiometric HfO_x_ layer is important for developing low-voltage operating devices.

The accurately measured concentration of V_o_ is essential for improving its controllability. However, the overproduction of V_o_ poses a challenge to the reliability of the device; i.e., an optimal amount of V_o_ must be generated. Therefore, techniques that can prevent excess V_o_ must be explored. Yong et al. examined the relationship between RS and physicochemical properties of a TiN metal BE. They compared two types of HfO_x_-based RS devices fabricated with TiN BEs and deposited them using PVD and ALD [215]. The HfO_x_ layer on the PVD-TiN electrode was more oxygen deficient than that on the ALD-TiN electrode. As the ALD-TiN electrode was fabricated with O_2_ plasma, the residual oxygen produced TiO_y_N_z_ on its surface. Meanwhile, the PVD-TiN electrode was deposited in a stronger vacuum; the vacuum chamber was not connected to any oxygen source, which resulted in an oxygen-deficient HfO_x_ layer on top. As illustrated in Fig. 11d, the PVD-TiN device exhibits smaller forming voltage values, which can be attributed to a higher concentration of V_o_ at the HfO_x_/PVD–TiN interface that effectively narrows the gap to be bridged for filament formation. Moreover, a higher initial defect density near the HfO_x_/PVD–TiN interface reduced filament base widening, which resulted in a thicker filament base than that of the HfO_x_/ALD–TiN interface. According to a simulation study by Carlo et al. [216], a thicker filament base at the bottom metal electrode interface can lead to progressive RESET behavior characterized by a gradual thinning of the CF and a moderate increase in the CF resistance. In addition, the larger area of the ruptured filament surface caused by a larger filament base resulted in a reduced memory window. Therefore, the formation of a thicker filament base at the HfO_x_/PVD–TiN interface led to gradual switching and a reduced memory window in PVD-TiN-based devices, as compared to the abrupt switching and large memory window caused by the sharp rupture in the thin CF formed within ALD-TiN devices. These studies indicated that the nature of constituent materials and deposition process influenced the V_o_ concentration at the interface of HfO_2_/electrode, and therefore, they played the definitive role of RS operation in HfO_x_-based RS devices.

#### Environment

During the switching operations, seamless gas exchange with the atmosphere improved the controllability of the redox reaction, which improved device performance. Kwon et al. demonstrated highly linear and symmetrical conductance modulation in HfO_2_-based RS devices by employing unique structures of HfO_2_ nanorods as a switching layer [217]. These nanorods enhanced the controllability of the redox reaction by permitting oxygen circulation between the oxide and ambient atmosphere (space between the nanorods) (Fig. 11e). The environment acted as a V_o_ reservoir and outlet for the emission of oxygen gas. Thus, this unique device structure precisely controlled the generation of V_o_ (oxygen ions) during RS operation, minimizing the randomness of the switching behavior.

## Conclusion and outlook

V_o_ exerts both positive and negative effects on the ferroelectricity of HfO_2_-based thin films. The positive effect is the suppression of the formation of stable non-ferroelectric monoclinic phase. Although oxygen deficiency is helpful in reducing the monoclinic phase fraction of HfO_2_-based thin films deposited using ALD or sputtering, as a crystallographic defect, it forms localized trap sites that deteriorate the insulation properties of the device by increasing the local conduction and nonideal local field near the V_o_. Moreover, the V_o_ retards the propagation of domain walls by decreasing the polarization switching speed, which is critical for maintaining the operation speed of ferroelectric memories. An increased V_o_ concentration enhances the relative stability and fraction of the non-ferroelectric tetragonal phase, which can degrade the ferroelectricity of HfO_2_-based thin films. Hence, an optimized V_o_ concentration range can help achieve both enhanced ferroelectricity and robust insulation free from charge trapping; moreover, the polymorphism of HfO_2_ can be controlled by the synergy of various factors including doping, thermal process, surface energy effect, and strain.

The three main types of ferroelectric memories categorized by their cell structure are as follows: (1) 1 transistor 1 capacitor ferroelectric random-access memories (FeRAM), (2) 1 transistor ferroelectric field-effect transistor (FeFET), and (3) 1 resistor ferroelectric tunnel junction (FTJ). Details of the ferroelectric memories with the different cell types are available in other reviews [218, 219]. Although the operation principles of the three devices are different, the properties they require to be considered as alternatives for state-of-the-art memory devices are similar: (1) high information density with ultra-large-scale integrated circuits with dimensional scalability, (2) high operation speed, (3) sufficient reliability including high switching endurance and low cycle-to-cycle variability, and (4) low device-to-device variability correlated to the spatial uniformity.

A high V_o_ concentration would be detrimental to achieving high information density with dimensional scaling. Such concentrations are frequently observed at interfacial regions near the electrodes or semiconductor channels. With decreasing film thickness for dimensional scaling, the increased fraction of the oxygen-deficient interfacial layers would result in an increased fraction of non-ferroelectric metastable phases, such as the tetragonal phase; the nonideal leakage current density through the ferroelectric HfO_2_ film would also increase. Moreover, a high concentration is not conducive to achieving high operation speeds, because it would decelerate the propagation of domain walls resulting from the attraction between defects. The frequently reported read-after-write latency [220, 221] is also strongly correlated to charge trapping/de-trapping, which is generally significantly slower than the polarization switching dominated by the domain wall propagation [222–225]. Furthermore, a high V_o_ concentration decreases the average grain size in oxide thin films. Therefore, the increased density of GBs is another cause of the decelerating switching speed of ferroelectric HfO_2_ thin films [31].

The main failure mechanism of the ferroelectric HfO_2_ is the hard breakdown that arises from the increased concentration and agglomeration of V_o_ with CFs forming in metal/ferroelectric/metal capacitors of FeRAM and FTJs or charge trapping and interfacial trap generation in the metal/ferroelectric/semiconductor gate stack of FeFETs. Therefore, neither mechanism should contain a high V_o_ concentration. The origin of the cycle-to-cycle variability with wake-up, fatigue, and soft or hard breakdown observed during repetitive polarization switching is also the change in V_o_ concentration and its drift, which results in its redistribution. Therefore, V_o_ concentration should be decreased to achieve reliable ferroelectric memories.

In addition to the V_o_ concentration, the spatial distribution of the concentration should also be controlled to decrease device-to-device variability. It should be also noted that numerous factors can affect the device-to-device variability of ferroelectric memory arrays, including polymorphism and film texture [226]. However, V_o_ distribution is a key factor that impacts this variability.

Therefore, the right strategy to achieve the optimized electrical properties of ferroelectric memories should be to decrease the V_o_ concentration by adequately controlling the other factors to suppress polymorphism, which is in turn achieved by suppressing the stable monoclinic phase formation. As described in the preceding paragraphs, the high V_o_ concentration has various detrimental effects on the material properties and the resulting device performance and reliability. However, the monoclinic phase fraction should be decreased by modulating factors other than the V_o_ concentration.

Meanwhile, it is necessary to consider the requirements of V_o_ concentration in RS phenomena from a different perspective. As previously reported, RS devices based on HfO_2_ employ a capacitor structure similar to that of FeRAM or FTJs, which possess a metal/RS layer/metal configuration. The equivalent of the failure mechanism in ferroelectric research is an electroforming process in RS research that triggers the subsequent RS behavior. Because the electroforming process does not contain an oxygen-deficient sub-phase, the RS behavior is determined solely by the generation, electromigration, and agglomeration of V_o_. The V_o_ dynamics are influenced by intrinsic defects and those generated under voltage applications, which results in the formation of a conductive pathway known as the CF; this CF enables seamless RS. This switching mechanism is called a VCM because the presence of V_o_ in the crystal lattice of HfO_2_ leads to a change in the valence state. The migration of these oxygen ions or vacancies changes the local stoichiometry of the switching layer, inducing a valence change in the cation sublattice and modifying the electronic conductivity. In VCM devices, the switching behavior is associated with various phenomena related to a CF’s evolution, which occur through the formation, migration, agglomeration, and rearrangement of V_o_. Once a CF has been formed, the movement of V_o_ becomes critical to the switching operation. Under an external electric field, V_o_ migrate within the switching layer and toward the positively biased electrode (anode) owing to electrostatic forces. This migration process leads to RESET (LRS to HRS) and SET (HRS to LRS) operations, which in turn results in the rupture and reformation of CFs.

Therefore, in addition to the concentration of V_o_, factors such as the mobility of oxygen ions and structural irregularities within the switching material play a crucial role in facilitating the migration and agglomeration of V_o_, affecting the RS performance. The concentration, migration, and agglomeration of intrinsic V_o_ defects are strongly influenced by crystallinity, stoichiometry, metal doping, interfacial layers, and electrode material. Particularly, in crystallized HfO_2_, GBs are considered active regions for RS or CF formation because they provide an environment where V_o_ can more easily form, migrate, and agglomerate than in bulk areas. The lower diffusion barrier for V_o_ at the GBs facilitates their movement and agglomeration. During the HfO_2_ deposition process, introducing certain metals as dopants can form oxygen-deficient regions owing to the availability of V_o_ and the ease of breaking metal-O bonds. The presence of V_o_ and the altered bonding contributes to the formation and stabilization of CFs. In addition, the selection of an appropriate interfacial layer or electrode can significantly influence the creation of an external reservoir for the V_o_. This reservoir increases the reliability of the device during repeated switching events. By acting as a source or sink for the V_o_, the interfacial layer or electrode helps maintain the stability and consistency of CF formation and rupture during the switching process. Therefore, understanding and controlling the GBs, stoichiometry, and appropriate interfaces/electrodes are important in the design and performance optimization of crystallized HfO_2_-based RS devices. Consequently, investigating those relationships has been a major focus of research. These factors are determined during the deposition and fabrication processes, as well as by post-processing and were detailed in this review. The critical parameters of RS devices—operating voltage, current, switching speed, gradual conductance modulation, endurance, device reliability, interdevice and intradevice variations, and long-term retention properties—can be optimized by precisely tuning these factors, which were also elaborately discussed.

**Fig. 12:**
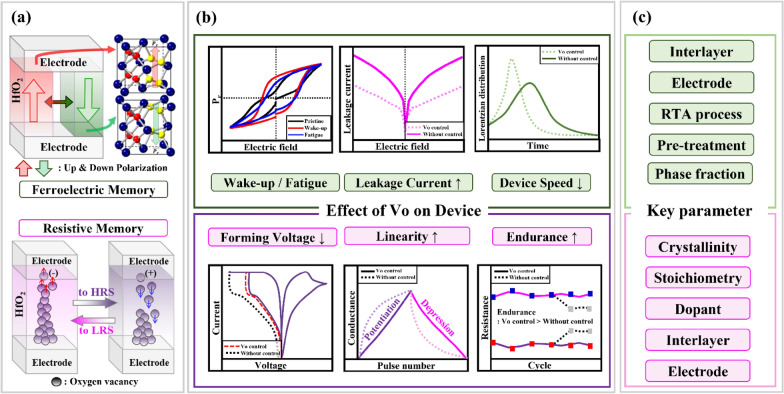
Comprehensive schematic of the ferroelectric and RS HfO_2_ memory device and the effect of V_o_ for their performances. **a** Ferroelectric switching mechanism based on the movement of oxygen atoms (up) and V_o_-mediated RS mechanism (down). **b** Effects of V_o_ and their roles in ferroelectric and resistive memory performance. **c** Key parameters affecting the concentration and motion of V_o_ in the film

Consequently, precise control over the V_o_ concentration in HfO_2_ can enhance both the ferroelectric properties and RS performances. As shown in Fig. 12, the concentration and migration of V_o_ may modify the properties of ferroelectric and RS devices, and it can be controlled by adjusting several key parameters. In the field of ferroelectric research, the main focus of studies thus far has been on the wake-up effect and fatigue behavior prior to soft or hard breakdown. Meanwhile, in RS research, the critical concern has been on comprehending the switching performance after a soft breakdown (CF formation). Due to these divergent objectives, controlling V_o_ by a unified method is not possible or necessary. Nevertheless, it is worthwhile to study the influence of factors on the generation, migration, and agglomeration of V_o_ during the deposition of HfO_2_ and fabrication of the device, and the effect on the structural, chemical, and, thus, electrical properties under voltage application. This insight can provide valuable guidance for future endeavors aimed at achieving desirable behaviors in both ferroelectric and RS properties.

## Data Availability

The review is based on the published data and sources of data upon which conclusions have been drawn can be found in the reference list.

## Abbreviations

RS: Resistive switching
V_o_: Oxygen vacancy
MOSFET: Metal oxide semiconductor field effect transistor
ALD: Atomic layer deposition
CSD: Chemical solution deposition
CMOS: Complementary metal-oxide semiconductor
HZO: Hf_0.5_Zr_0.5_O_2_
PLD: Pulsed laser deposition
CF: Conductive filament
DRAM: Dynamic random-access memory
TEM: Transmission electron microscope
3D: Three-dimensional
ToF–SIMS: Time-of-flight secondary ion mass spectroscopy
O_2_^*^: O_2_ plasma
HAABF: High-angle annular bright-field
TALD: Thermal atomic layer deposition
RPALD: Remote plasma atomic layer deposition
DPALD: Direct plasma atomic layer deposition
XPS: X-ray photoelectron spectroscopy
DFT: Density functional theory
VASP: Vienna ab initio simulation package
RF: Radio frequency
RTA: Rapid thermal annealing
ΔH_f_ per O: Enthalpy per oxygen of 1 mol
RRAM: Resistive random-access memory
HRS: High resistance state
LRS: Low resistance state
HX-PES: Hard X-ray photoelectron spectroscopy
HAADF-STEM: High-angle annular dark-field scanning transmission electron microscopy
iDPC-STEM: Integrated differential phase contrast scanning transmission electron microscopy
LSMO: La_0.67_Sr_0.33_MnO_3_
FFT: Fast Fourier transform
EELS: Electron energy loss spectroscopy
EDS: Energy dispersive X-ray spectroscopy
HR-TEM: High-resolution transmission electron microscope
EFM: Electrostatic force microscopy
CB: Conduction band
c-AFM: Conductive atomic force microscopy
2D: Two-dimensional
TE: Top electrode
BE: Bottom electrode
STXM: Scanning transmission x-ray microscopy
GB: Grain boundary
NEB: Nudged elastic band
PF: Poole–Frenkel
TAT: Trap-assisted tunneling
FN: Fowler–Nordheim
GIXRD: Grazing incidence X-ray diffraction
2P_r_: Double remanent polarization
E_c_: Coercive field
FORC: First-order reversal curve
E_bias_: Internal bias
VO_x_: Vanadium oxide
VCM: Valence change mechanism
ECM: Electrochemical metallization
MBE: Molecular beam epitaxy
RMBE: Reactive molecular beam epitaxy
PVD: Physical vapor deposition
FeRAM: Ferroelectric random-access memory
FTJ: Ferroelectric tunnel junction
FeFET: Ferroelectric field effect transistor
